# Epigenetic regulation in transplant rejection and tolerance: mechanisms and therapeutic prospects

**DOI:** 10.3389/fcvm.2026.1878170

**Published:** 2026-08-21

**Authors:** Yuhao Li, Shuan Ran, Yanqiang Zou, JiKai Cui

**Affiliations:** 1 Department of Cardiovascular Surgery, Union Hospital, Tongji Medical College, Huazhong University of Science and Technology, Wuhan, China; 2 Center for Translational Medicine, Union Hospital, Tongji Medical College, Huazhong University of Science and Technology, Wuhan, China; 3 NHC Key Laboratory of Organ Transplantation, Ministry of Education, Chinese Academy of Medical Sciences, Wuhan, China

**Keywords:** acute rejection, biomarker, chronic rejection, epigenetics, graft-vs.-host disease, ischemia–reperfusion injury, transplant rejection, transplant tolerance

## Abstract

Epigenetics modulates gene expression and plays a pivotal role in numerous immune disorders. Over the past two decades, increasing evidence has indicated that epigenetic regulation contributes to transplant rejection and immune tolerance. Four primary epigenetic mechanisms—DNA methylation, histone modifications, chromatin remodeling, and non-coding RNAs (ncRNAs)—exert distinct effects on rejection after transplantation. Therefore, understanding the epigenetic mechanisms of rejection provides novel avenues for inducing immune tolerance and developing strategies to prolong graft survival and patient longevity. This review outlines the history of epigenetics and highlights landmark events since the term was coined. Furthermore, we summarize epigenetic research methodologies and describe the four primary general mechanisms of epigenetic regulation. We subsequently focus on recent studies supporting the role of epigenetic regulation in distinct pathological processes of solid organ transplantation (namely, ischemia‒reperfusion injury, acute rejection, and chronic rejection) and graft-vs.-host disease following hematopoietic stem cell transplantation. Finally, we discuss the clinical applications of epigenetics in transplant rejection, including predictive diagnostics and the induction of immune tolerance, to guide the future development of improved rejection suppression therapies.

## 1 Introduction

As the primary treatment for end-stage organ failure, including advanced heart disease, organ transplantation still faces the significant challenge of transplant rejection in achieving long-term success. In 2024, a total of 173,727 organ transplants were performed globally, with the United States and China ranking first and second, respectively, at 48,935 and 24,684 cases (1). Although the clinical application of immunosuppressive agents has significantly improved short-term patient survival rates, issues such as chronic rejection, drug toxicity, and secondary infections remain fundamentally unresolved (2, 3). The traditional immunological perspective has focused primarily on T-cell- and B-cell-mediated adaptive immune responses. However, increasing evidence has indicated that epigenetic regulatory mechanisms play crucial roles in directing the activation, differentiation, and memory formation of immune cells (4–7).

Epigenetic regulation occurs at multiple levels, including DNA methylation, histone modification, chromatin remodeling, and non-coding RNA regulation (8–10). Epigenetic modifications dynamically regulate gene transcription without altering DNA sequences, providing new insights into the “plasticity” and “memory” of immune responses. Epigenetic markers serve as important molecular indicators of transplant rejection, aiding in the diagnosis of ischemia‒reperfusion injury and transplant rejection (11, 12). Furthermore, these modifications can be harnessed therapeutically by modulating Tregs and DCs to induce immune tolerance (13). Although much of the current evidence stems from studies on kidney, liver, lung, and hematopoietic stem cell transplants, these studies have identified conserved epigenetic mechanisms that are highly relevant to heart transplantation. Emerging heart-specific evidence further suggests that epigenetic regulation is involved in the development of rejection in heart transplantation, highlighting its potential for improving long-term graft survival. Therefore, this review synthesizes evidence across transplantation fields while emphasizing its implications for heart transplantation.

## 2 Overview of epigenetics

### 2.1 A brief history of epigenetics

Since C.H. Waddington first introduced the revolutionary concept of “epigenetics” in 1942, research in this field has flourished (14). Early foundational discoveries, such as Hotchkiss et al.'s 1948 paper on analyzing DNA using paper chromatography (15) and Razin et al.'s 1980 report of the inhibitory effects of DNA methylation (16). Concurrently, research into histone modifications has made significant progress. In 1964, histone acetylation was first identified and found to be closely linked to the regulation of RNA synthesis (17). Moreover, Sanger first identified circRNA molecules in viroids (18) and the first lncRNA was identified as H19 (19). In 1993, Lee et al. discovered the first microRNA, lin-4 (20), confirming the pivotal importance of small RNAs in gene silencing.

In recent decades, the application of epigenetics in immunology has become particularly prominent. In 1998, researchers discovered that changes in the methylation of the *IL-4*, *IL-13*, and *IFN-γ* genes significantly influence the differentiation of naive T cells into Th1 and Th2 subsets (21, 22). Years later, Lee et al. further demonstrated that DNA methylation and DNA Methyltransferase 1 (DNMT1) were crucial for the proper maturation of thymic progenitor cells (23). Meanwhile, research indicated that the clonal expression patterns of KIRs in NK cells are maintained through DNA methylation (24, 25). In 2006, Fann and colleagues discovered that histone H3K9 hyperacetylation may participate in the selective and rapid gene expression of memory CD8⁺ T cells (26). Foxp3, a forkhead transcription factor that is specifically expressed in Tregs to promote graft survival, undergoes fine-tuned developmental and functional regulation by DNA methylation and histone acetylation (27–30). In 2007, Santa et al. reported that Jmjd3 profoundly influences macrophage transdifferentiation by binding to target genes and regulating H3K27me3 levels (31).

**Figure 1:**
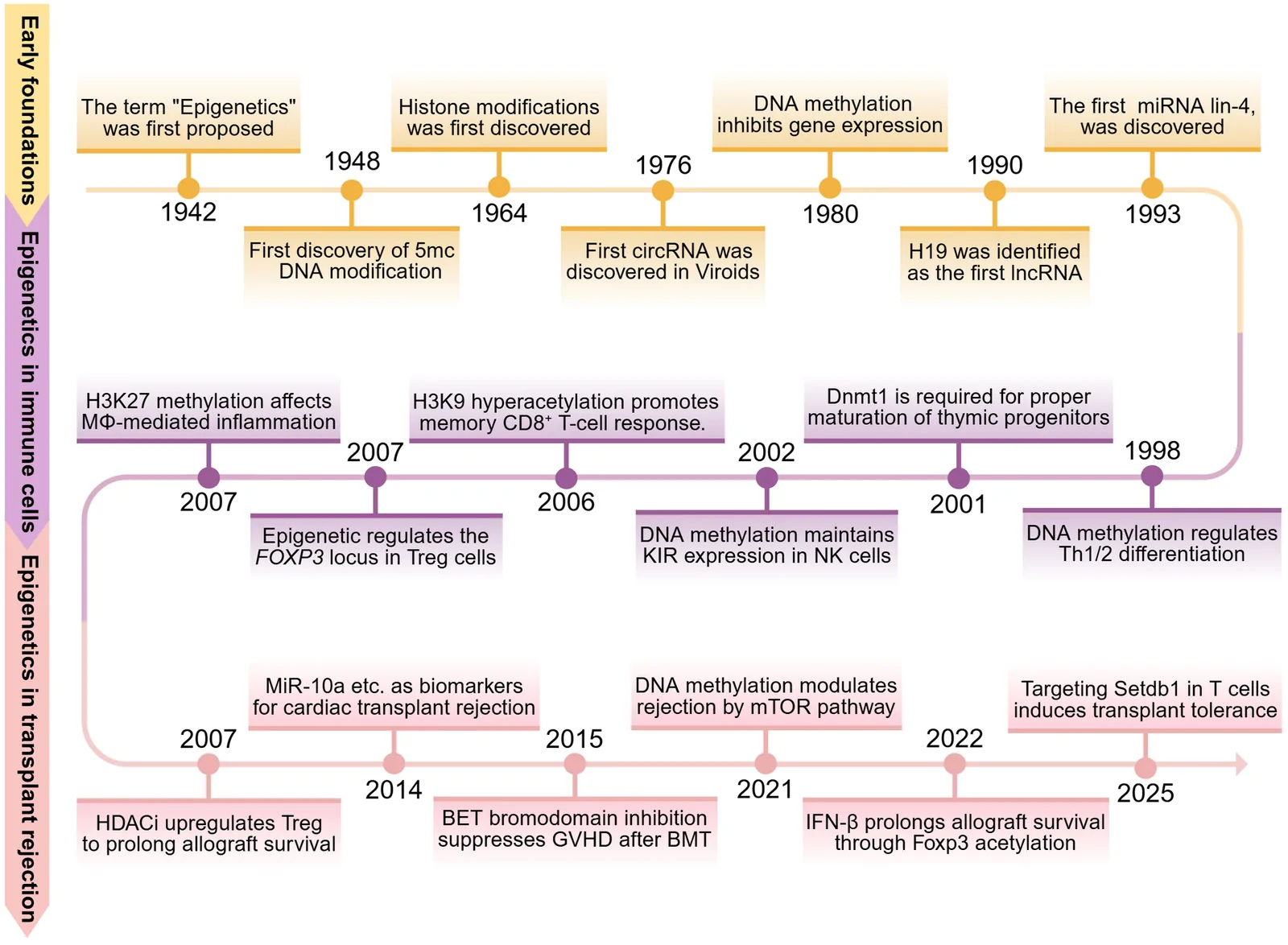
Development of epigenetics and significant historical advances in understanding its roles in immune cells and rejection responses. This figure is divided into three sections: the historical development of early epigenetics research, epigenetic regulation within immune cells, and epigenetic mechanisms or applications in transplant rejection.

In the 21st century, the role of epigenetics in transplant immunology has become increasingly prominent. In 2007, Tao et al. demonstrated that *in vivo* HDACi therapy enhances Treg-mediated steady-state suppression of proliferation (32). It is also important to indicate the occurrence of rejection, Huyan et al. reported that the expression levels of four specific miRNAs highly distinguished the occurrence of acute rejection in heart transplant (33). In 2015, Sun et al. observed that using I-BET151 significantly regulates cytokine expression in DCs and T cells and alleviates graft-vs.-host disease (34). In 2021, Zhu et al. discovered that DNA methylation levels influence acute rejection by regulating mTOR signaling (35). In 2022, Francisco et al. reported that IFN-*β* increases Treg induction via Foxp3 acetylation (36). Looking ahead, a 2025 study using a mouse heart transplant model suggested that targeting Setdb1 in CD4⁺ T cells may represent an effective strategy for inducing transplant tolerance (37) (Figure 1).

## 3 Epigenetic regulatory mechanisms

**Figure 2:**
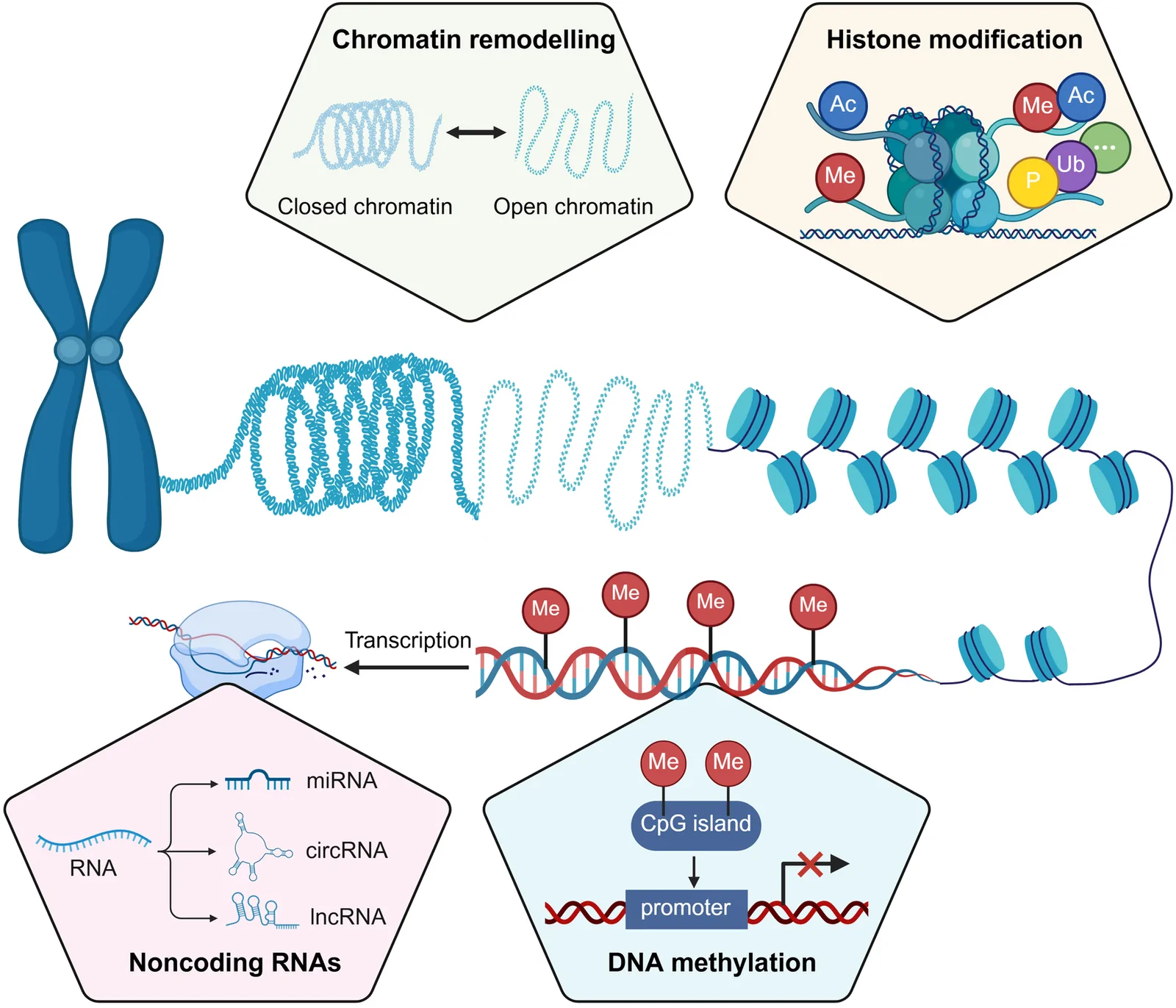
Four general epigenetic regulatory mechanisms. This diagram illustrates DNA methylation, histone modifications, chromatin remodeling, and ncRNAs. DNA methylation is a common chemical modification that typically involves the addition of methyl groups (Me) to CpG islands within DNA molecules. Histones undergo several distinct posttranslational modifications, including acetylation (Ac), methylation (Me), phosphorylation (P), and ubiquitination (Ub). Chromatin remodeling complexes alter chromatin packaging by moving, sliding, disrupting, or rearranging nucleosomes. ncRNAs participate in diverse cellular processes by targeting various molecules (e.g., functioning as molecular sponges).

We discuss four principal mechanisms of epigenetic regulation: DNA methylation, histone modifications, chromatin remodeling, and non-coding RNAs (Figure 2).

### 3.1 DNA methylation

DNA methylation is a prevalent chemical modification in eukaryotic cells and is the primary epigenetic mechanism that regulates the mammalian genome. This modification encompasses various forms, including 5-methylcytosine (5mC), 5-hydroxymethylcytosine (5hmC), 5-formylcytosine (5fC), and 5-carboxylcytosine (5caC) (38–40). While these modifications can occur throughout the genome, they are predominantly found within cytosine‒phosphate‒guanine (CpG) islands. Among these modifications, 5mC is the most abundant epigenetic modification in the human genome.

DNA methylation is a dynamic process. DNA methyltransferases (DNMTs) catalyze the transfer of methyl groups from S-adenosylmethionine to cytosine residues. DNMTs are broadly categorized into two functional groups: *de novo* DNMTs (DNMT3A and DNMT3B) and maintenance DNMTs (DNMT1). During DNA replication, DNMT1 binds to nascent DNA and methylates it, precisely replicating the methylation pattern present prior to replication (41). DNMT3A and DNMT3B are capable of methylating naked DNA and establishing novel methylation patterns on unmodified DNA templates (42).

DNA demethylation occurs via two principal mechanisms: passive and active. Passive demethylation occurs in proliferating cells, where the inhibition or dysfunction of DNMT1 prevents the methylation of newly synthesized DNA strands. Active demethylation involves the conversion of 5mC to 5hmC by TET dioxygenases. Two distinct pathways can reverse 5hmC to cytosine. The first pathway involves iterative oxidation reactions mediated by the ten-eleven translocation (TET) protein family (43). The second pathway involves the deamination of 5hmC by AID/APOBEC proteins, though this pathway does not play a universal role in active demethylation processes in mammals (44).

DNA methylation is recognized by three distinct protein families: MBD proteins, UHRF proteins, and zinc finger proteins. MBD proteins possess a conserved MBD that has high affinity for methylated CpG sites (45, 46). UHRF proteins comprise UHRF1 and UHRF2, specifically UHRF1 maintains DNA methylation by interacting with DNMT1 and directing it to half-methylated DNA regions (47, 48). The zinc finger protein family inhibits transcription in a DNA methylation-dependent manner (49).

### 3.2 Histone modification

Histone modifications, a class of posttranslational modifications, regulate chromatin structure—altering its loose or compact state. Common histone modifications include acetylation, methylation, phosphorylation, and ubiquitination (50, 51). Among these modifications, histone acetylation and methylation have been investigated most extensively.

#### 3.2.1 Histone acetylation

Histone acetylation predominantly occurs at the N-terminal lysine residues of histones H3 and H4. This modification is governed primarily by histone acetyltransferases (HATs) and histone deacetylases (HDACs). Acetylation neutralizes the positive charge of lysine, potentially weakening the electrostatic interactions between positively charged histones and negatively charged DNA. The primary HAT families include GCN5-associated N-acetyltransferases, the MYST family, and the CBP/P300 complex (52, 53). The HDAC family is classified into four classes based on structural similarity and substrate specificity. Notably, Class III (sirtuins 1–7) are nicotinamide adenine dinucleotide-dependent deacetylases (54). Acetylation is recognized by histone acetyltransferase readers, primarily bromodomain proteins.

#### 3.2.2 Histone methylation

Histone methylation predominantly occurs at the N-terminal lysine or arginine residues of histones H3 and H4 and is governed by histone methyltransferases (HMTs) and histone demethylases (HDMs). Histone lysine methyltransferases and histone arginine methyltransferases respectively transfer methyl groups from SAM to the *ε*-amino group of lysine or the *ω*-guanidino group of arginine. Lysine can be monomethylated, dimethylated, or trimethylated, whereas arginine can exist in monomethylated, symmetric dimethylated, or asymmetric dimethylated forms (55, 56). Methylation of distinct histone sites has various effects. For instance, methylation of H3K4, H3K36, and H3R17 is generally associated with transcriptional activation (57–59). Conversely, methylation of H3K9, H3K27, and H4K20 is linked to transcriptional repression (60–62).

### 3.3 Chromatin remodeling

Chromatin remodeling is a fundamental mechanism that regulates gene expression by altering the chromatin structure. Nucleosomes, consisting of DNA fragments wrapped around histone octamers, impede gene transcription (63). Chromatin remodeling complexes employ ATP hydrolysis to displace nucleosomes or modulate histone‒DNA interactions, thereby controlling the compactness and functional state of the chromatin fiber (64). This process directly determines whether transcription factors and other molecules can bind to target DNA by precisely regulating the accessibility of specific genomic regions.

Chromatin remodeling complexes are classified into four families: SWI/SNF, ISWI, inositol (INO80), and chromodomain helicase DNA binding (CHD) (65, 66). The SWI/SNF complex comprises three main modules: the ATPase module, the ARP module, and the core module (67). The ISWI family complex also consists of three parts: an N-terminal RecA-like helicase domain, a C-terminal HAND-SANT-SLIDE domain, and a regulatory autoinhibitory domain (68). Similar to SWI/SNF, INO80 is composed of an ATPase domain, ARP, and a core module (69). The CHD family of remodeling complexes interacts with factors involved in chromatin extension and modification (70).

### 3.4 Non-coding RNAs

Non-coding RNAs are RNA molecules that do not encode proteins and encompass a diverse set of functional RNAs, including ribosomal RNA (rRNA), transfer RNA (tRNA), microRNA (miRNA), small nucleolar RNA (snoRNA), small interfering RNA (siRNA), etc. (71). Based on their length, ncRNAs can be broadly categorized into small ncRNAs (18–200 nucleotides), lncRNAs (>200 nucleotides), and circRNAs. Furthermore, sncRNAs can also be divided into miRNAs, snoRNA, siRNA, etc. We will focus on discussing the structure and function of miRNAs, lncRNAs and circRNAs.

#### 3.4.1 MiRNAs

MicroRNAs are a class of endogenous small RNAs. The transcription of most miRNA genes is initiated by RNA polymerase II (Pol II), generating large primary miRNA transcripts (pri-miRNAs) which form characteristic hairpin structures (72). Within the nucleus, pri-miRNA is cleaved by Drosha into the pre-miRNA. After export of pre-miRNAs to the cytoplasm, they are cleaved into small dsRNAs (one is guide and the other is passenger strand) by Dicer. Subsequently, the miRNA double strands are loaded onto the argonaute protein. Upon restoring AGO to its native conformation, the passenger strand of the miRNA double-stranded RNA is ejected, yielding a single-stranded mature miRNA. miRNAs primarily suppress gene expression and translation by forming base pairs with the 3′-UTR of target mRNAs (73). The dysregulation of miRNAs is implicated in the pathogenesis of various diseases, including cancers, metabolic disorders and immune-mediated diseases (74–76).

#### 3.4.2 LncRNAs

LncRNAs constitute a diverse family of non-coding RNAs exceeding 200 nucleotides in length. Based on their genomic location, lncRNAs can be broadly classified into five major categories: sense overlapping RNAs, antisense RNAs, long intergenic non-coding RNAs, sense intron RNAs, and processed non-coding RNAs. lncRNAs regulate gene expression patterns through various molecular mechanisms (77). lncRNAs exhibit remarkable functional specificity because they participate in a wide array of cellular processes, notably DNA methylation and histone modifications (78).

#### 3.4.3 CircRNAs

CircRNAs represent a diverse class of endogenous non-coding RNAs distinguished by their covalently closed circular topology, lacking free 3′ and 5′ termini. This circularization process depends on the specific RNA source material (79, 80). Based on their biogenesis and composition, circRNAs can be broadly categorized into four main types: exon circRNAs, intron circRNAs, exon‒intron circRNAs, and fusion circRNAs (81). circRNAs can specifically bind to microRNAs via complementary binding sites, thereby sequestering and inhibiting miRNA activity (82). Furthermore, circRNAs can interact with RNA-binding proteins and function as transcriptional regulators (83, 84).

## 4 Classification of rejection

Organ transplantation is accompanied by a cascade of pathophysiological alterations from the donor to the recipient. Ischemia‒reperfusion injury represents an early and critical event, as grafts endure cold ischemia during hypothermic preservation, warm ischemia during cardiac arrest and surgical manipulation, and reperfusion injury upon the restoration of blood flow (85). Furthermore, allograft rejection remains a significant impediment to graft survival.

**Figure 3:**
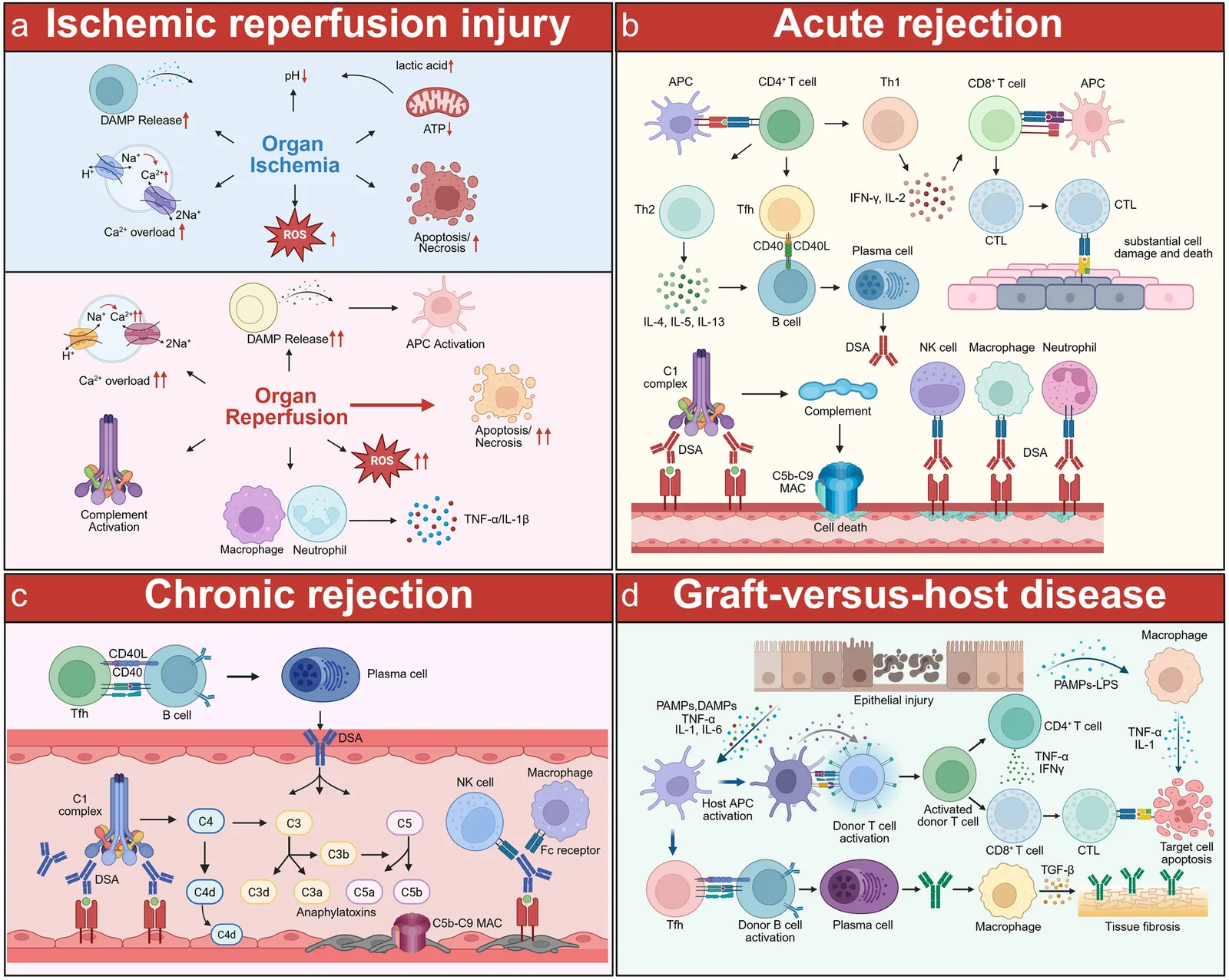
Pathophysiological mechanisms in transplant immunology. **(a)** Ischemia‒reperfusion injury. Ischemia induces DAMP release, Ca^2^⁺ overload, and increased ROS production, leading to mitochondrial dysfunction, ATP depletion, and acidosis. Reperfusion exacerbates damage by increasing oxidative stress and immune cell activation (complement, neutrophils), increasing cell death. **(b)** Acute rejection. Donor Class I/II MHCs on APCs engage recipient T cells, differentiating them into effector cells (Th1, Th2, and CTLs). CTLs eliminate graft cells via perforin/granzyme and Fas/FasL signaling. Activated B cells produce DSAs, promoting complement-mediated MAC and innate cell recruitment. **(c)** Chronic rejection. B cells secrete DSAs, which activate the classical complement pathway and promote MAC formation. This process recruits macrophages, NK cells, and effector cells that target the vascular endothelium. **(d)** Graft-vs.-host disease (GvHD). PAMPs/DAMPs from damaged host cells activate host APCs. These cells present donor antigens to donor immune cells, causing target cell apoptosis and fibrosis.

HVGR (clinically referred to as rejection) describes the response of the recipient's immune system to the donor allograft. Based on the temporal kinetics and pathological features, HVGR can be further stratified into hyperacute rejection, acute rejection, and chronic rejection (86). In contrast, GVHR involves immune-mediated attacks on recipient tissues by donor immune cells, typically occurring in the context of hematopoietic stem cell transplantation (Figure 3).

Hyperacute rejection manifests within minutes to hours after transplantation. It is mediated by a rapid humoral immune response triggered by pre-existing recipient antibodies directed against donor tissue antigens. Fortunately, current pretransplant anti-donor antibody screening protocols have effectively mitigated the incidence of hyperacute rejection. Therefore, our subsequent discussion focuses on the molecular mechanisms, pathological alterations, and associated epigenetic regulatory processes governing IRI, AR, CR, and GVHD.

### 4.1 Ischemia‒reperfusion injury

IRI refers to the phenomenon in which the reperfusion to an organ or tissue previously subjected to a period of ischemia and hypoxia results in cellular dysfunction, exacerbated apoptosis/necrosis, and both local and systemic inflammatory responses.

During the interval between procurement of the donor organ and post-implantation reperfusion, the organ is subjected to ischemia and profound hypoxia. This shift generates substantial quantities of lactate and H⁺ ions, precipitating intracellular acidosis (87). Consequently, intracellular ATP synthesis decreases drastically, leading to substantial cellular energy depletion (88). Dysfunction of the Na⁺/K⁺ pump results in intracellular Na⁺ retention, which drives a significant influx of Ca^2^⁺ via the Na⁺/Ca^2^⁺ exchanger, ultimately causing calcium overload (89). During ischemia, ATP catabolism results in the accumulation of hypoxanthine, which, in the presence of xanthine oxidase, generates hydrogen peroxide (90). Concurrently, the cessation of blood flow triggers mechanotransduction pathways, activating NADPH oxidase to produce ROS (91). The damage caused by ischemia ultimately culminates in cell death, accompanied by the release of DAMPs (92).

During reperfusion, oxygen is incompletely reduced by various intracellular enzymes, leading to the generation of substantial amounts of ROS (93). These ROS attack biological membranes, inducing lipid peroxidation (94). Furthermore, ROS impair ion pump functions, thereby exacerbating calcium influx. Upon continuous stimulation, vascular endothelial cells become activated, upregulating the expression of adhesion molecules. This activation results in the recruitment and activation of neutrophils and other inflammatory cells to infiltrate tissues and secrete additional ROS, proteases, and proinflammatory cytokines (95, 96). DAMPs stimulate the complement system, leading to the formation of MACs and generation of anaphylatoxins (97).

Ischemia–reperfusion injury predominantly causes parenchymal cell apoptosis and necrosis, in addition to damage to the microvascular system. For instance, characteristic features of hepatic IRI injury include hepatocyte edema, ballooning degeneration, and necrosis, with cholestasis in the bile ducts being the most prominent manifestation (98).

### 4.2 Acute rejection

Acute rejection represents the most frequent form of rejection of allogeneic organ transplants, typically manifesting within days to two weeks after transplantation. While this reaction gradually weakens after three months, a certain risk remains and is usually managed with immunosuppressive drugs. Acute rejection is predominantly mediated by cellular rejection, leading to the death or apoptosis of the parenchymal cells of a transplanted organ.

Acute cellular rejection is most commonly T-cell-mediated. But not exclusively, mediated by T cells, as other immune components may also contribute. This process involves two distinct pathways for allogeneic recognition: direct and indirect recognition (99). In the direct recognition pathway, recipient T cells directly engage antigen‒peptide complexes presented on donor APCs via allogeneic MHC molecules. This pathway represents a unique antigen presentation and recognition mechanism in allogeneic transplantation (100). In the indirect pathway, recipient T cells recognize allogeneic antigen peptides that have been processed and presented by self-APCs in conjunction with self-MHC molecules (101).

Following activation, CD4⁺ T cells differentiate into Th1, Th2, Th17, and Treg subsets (102, 103). Th1 cells produce cytokines such as IFN-*γ*, which recruit monocytes/macrophages, promote NK cell activation (104, 105). Th2 cells secrete cytokines such as IL-4 and interact with B cells, promoting B-cell proliferation (106–108). Th17 cells generate cytokines such as IL-17, which stimulate the production of inflammatory cytokines and chemokines, leading to the recruitment of neutrophils and macrophages to the transplanted organ (109, 110). Concurrently, activated CD8⁺ T cells differentiate into CD8⁺ CTLs. These CTLs become activated through the recognition of donor MHC class I molecules and the action of Th1-secreted IL-2/IFN-*γ* (111). CD8⁺ CTLs induce apoptosis or necrosis of parenchymal cells by releasing perforin and granzyme and via Fas/FasL pathway (112, 113).

Acute rejection mainly leads to parenchymal cell necrosis within tissues and organs, accompanied by lymphocyte and macrophage infiltration. In accordance with the ISHLT criteria, acute rejection of heart transplants primarily manifests as lymphocyte infiltration, myocardial cell injury and necrosis, and capillary endotheliitis (114).

### 4.3 Chronic rejection

Chronic rejection refers to the progressive deterioration of a patient's allograft, culminating in end-stage organ failure. This process typically manifests years and represents the principal impediment to long-term graft survival.

IRI during transplantation and other nonantigen-specific insults may influence chronic rejection (115, 116). In solid organ transplantation, AMR is considered the primary mechanism associated with chronic rejection. In AMR, B cells bind to alloantigens and, under the influence of Th2 and Tfh cells, differentiate into plasma cells that produce donor-specific antibodies (DSAs). DSAs form antigen‒antibody complexes with MHC antigens, activating the complement system (117). The complement-derived MAC can directly lyse target cells (118). Concurrently, released complement fragments exacerbate local graft inflammation through receptor-mediated chemotaxis and the activation of neutrophils and macrophages (119, 120). The antigen‒antibody complexes recruit NK cells and neutrophils to mediate target cell destruction (121, 122).

Activated immune cells release proinflammatory cytokines and chemokines, especially TGF-*β*. Under the influence of TGF-*β*, vascular endothelial cells undergo the endothelial-to-mesenchymal transition (EndMT), acquiring fibroblast-like characteristics and secreting ECM (123). Concurrently, recruited macrophages polarize toward the M2 phenotype and secrete large amounts of profibrotic factors, which further increase myofibroblast activation and ECM synthesis. Moreover, the activity of matrix metalloproteinases is inhibited by TGF-*β* and other factors, leading to substantial deposition of ECM within organ tissues (124, 125). The vascular endothelium progressively thickens, resulting in luminal narrowing and organ ischemia. Ultimately, functional cells are extensively replaced by scar tissue, incapacitating the organ and leading to end-stage failure.

Chronic rejection is characterized by fibrous proliferation and vascular smooth muscle cell hyperplasia. Key pathological features of chronic renal rejection include glomerular and arterial lesions, along with interstitial fibrosis (126). Glomerular lesionsmanifest as a “double-track” appearance of the glomerular capillary loop basement membrane. The arterial intima undergoes diffuse thickening, often described as “onion-skin” concentric fibrosis.

### 4.4 Graft-vs.-host disease

GVHR is an immune response initiated by donor immune cells within an allogeneic bone marrow or hematopoietic stem cell transplant.

Acute GVHD typically manifests within 100 days after transplantation and occurs in the skin, gastrointestinal tract and liver (127–129). It is initiated by tissue damage, generating proinflammatory cytokines. This process increases the expression of MHC antigens and cell surface adhesion molecules (130). The resulting inflammatory signals activate APCs, directing donor allogeneic T cells to differentiate into various effector T-cell subsets and produce cytokines (131, 132). Concurrently, CD8^+^ T cells differentiate into CTLs that exert the primary cytotoxic effect. Activated macrophages also contribute by secreting inflammatory cytokines, including TNF-*α* and IL-1. These cytokines activate DCs, enhance allogeneic antigen presentation, and promote cell death and necrosis, thereby damaging the tissue architecture.

Chronic GVHD is a syndrome that typically presents more than 100 days after transplantation and it can present as lichen-type features, dryness, strictures or sclerosis affecting multiple organ systems. The initial stage of cGVHD commences with damage to host tissues induced by the subsequent release of inflammatory cytokines. The second stage is defined by thymic damage, which permits the emergence and selection of self- and allogeneic reactive T-cell populations and the loss of central and peripheral immune tolerance (133). Tfh cells subsequently influence B-cell activation, leading to the production of abundant autoantibodies targeting host tissue antigens. The terminal stage is characterized by macrophage activation, resulting in the secretion of TGF-*β* and subsequent fibroblast/myofibroblast activation. Activated fibroblasts/myofibroblasts synthesize and secrete copious amounts of extracellular matrix components, leading to ECM accumulation and fibrosis.

The skin, the most common and earliest site of aGVHD, typically manifests with vacuolization of basal layer cells and keratinocyte apoptosis (134). Hepatic cGVHD resembles primary biliary cholangitis, with the progressive destruction and loss of interlobular bile ducts (135).

## 5 Epigenetic regulatory mechanisms in transplantation

Given the clinical relevance of rejection and the existing evidence, we shall focus on discussing the mechanisms of action of DNA methylation, histone methylation, histone acetylation, miRNAs, lncRNAs, and circRNAs in rejection responses.

### 5.1 DNA methylation

**Figure 4:**
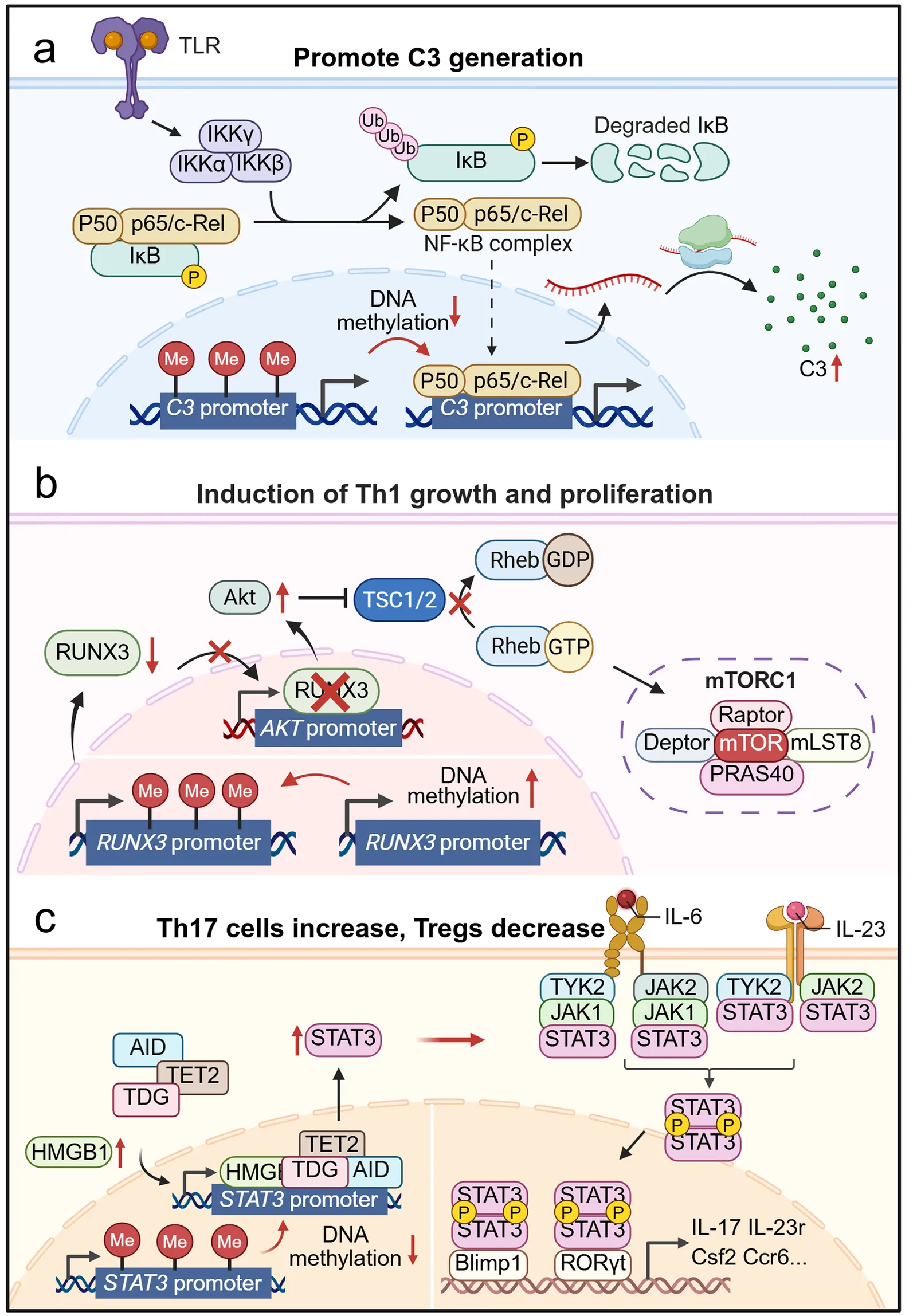
The role of DNA methylation in transplant immunology. **(a)** Induction of complement component 3 (C3) production. Reduced DNA methylation in the *C3* promoter region during ischemia–reperfusion injury activates the NF-*κ*B signaling pathway, increasing C3 expression. **(b)** Induction of Th1 growth and proliferation. Increased methylation of RUNX3 leads to its downregulation, resulting in increased Akt expression. Akt subsequently promotes Th1-mediated immune responses by activating downstream mTORC1 signaling pathways, thereby exacerbating acute rejection. **(c)** Increased numbers of Th17 cells, reduced numbers of Tregs, and the Th17/Treg imbalance. HMGB1 recruits DNA demethylases, including TET2, AID, and TDG, to form complexes at the *STAT3* promoter region, promoting the hypomethylation of the *STAT3* promoter in CD4^+^ T cells. This process leads to abnormal overexpression of STAT3, which subsequently activates inflammatory signaling pathways to increase cytokine expression (e.g., IL-17), thereby promoting Th17 differentiation while suppressing Treg differentiation.

DNA methylation profoundly influences gene accessibility and transcriptional activity by altering the nucleotide composition of DNA sequences (Figure 4).

#### 5.1.1 Ischemia–reperfusion injury

In ischemia-reperfusion injury, the damaging role of complement components is indispensable. In a rat model of kidney transplantation, Pratt et al. investigated the demethylation of CpG island within the IFN-*γ* response element in the promoter region of the rat *complement component 3* gene (136) (Figure 3a). In normal kidneys, 62% of CpG sites were observed to be methylated, a proportion that decreased to 42% and 31% following 24 h of cold ischemia and subsequent reperfusion, respectively. In addition to direct injury, tissue fibrosis is also a significant consequence of ischaemia-reperfusion injury. Genome-wide DNA methylation analysis reveals that severe IRI induces significant hypermethylation of the *LHX1* promoter in kidney progenitor cells (KPCs) (137). 8 DMRs hypermethylated and only 1 DMR hypomethylated in severe IRI KPCs, enriched in genes associated with transcription factors and other regulatory proteins. Moreover, as warm ischemia time increased, methylation levels rose from less than 50% to nearly 80%. The aforementioned epigenetic alterations have been validated in independent clinical cohorts through differential methylation analysis of *LHX1* using pyrophosphate sequencing. This study demonstrates that silencing caused by hypermethylation of the *LHX1* promoter compromises the kidney's self-repair capacity.

#### 5.1.2 Acute rejection

T cells are the primary effectors in acute rejection reactions, and DNA methylation modifications influence T cell function. Zhu et al. identified significant differences in genome-wide methylation patterns, detecting 1,021 DMRs, among which the *RUNX3* promoter region was the most hypermethylated DMR in kidney transplant recipients with AR (35). Simultaneously, a smaller target methylation region was selected for more detailed examination, revealing that this region overlaps with the first exon and the TSS200 region. To verify the hypermethylation of this target region, BSP and additional human PBMC samples from graft dysfunction/stable cohorts were analyzed, revealing that this target region was indeed much more hypermethylated. Mechanistically, RUNX3 functions as a negative regulator of the mTOR pathway (138); its reduced expression leads to Akt activation and the subsequent exacerbation of proinflammatory Th1, Th2, and Th17 responses through mTORC1 signaling (139, 140) (Figure 3b).

#### 5.1.3 Chronic rejection

The regulatory role of DNA methylation in the fibrotic response during CR has garnered increasing attention. In transplant-associated vascular pathology in humans and mice, it has been observed that suppression of TET2 expression and activity in arterial smooth muscle cells (VSMCs) within graft-associated vascular lesions of both human and murine recipients, with IFN-*γ* identified as a potential mediator (141). It has previously been reported that TET2 promoted 5hmC modification of the promoters of key VSMC differentiation genes. Further findings revealed that TET2 is a critical negative regulator of VSMC apoptosis in graft arterial lesions, highlighting its role in conferring protection against apoptosis under conditions of transplant vascular injury or IFN-*γ* stimulation. Importantly, high-dose vitamin C supplementation increased TET2 activity and ultimately ameliorated chronic rejection. Complementing these findings, multiomics analyses of kidney transplants have illuminated distinct DNA methylation landscapes associated with graft outcomes. Analysis of biopsy samples collected 24 months post-transplant from the interstitial fibrosis/tubular atrophy (IF/TA) group and the non-fibrotic/non-atrophic (NFA) group revealed an enrichment of hypomethylated CpG sites in the IF/TA group's biopsy samples, which was associated with inflammation; conversely, hypermethylation was associated with impaired renal repair (142). In parallel, a genome-wide DNA methylation analysis of PBMCs from kidney transplant recipients demonstrated that transplant tolerance was linked to the hypomethylation of genes critical for immune function (e.g., T/B-cell activation and Th17 differentiation). Conversely, chronic rejection is characterized by hypomethylation of genes involved in intracellular signaling and ubiquitination pathways (143).

#### 5.1.4 Graft-vs.-host disease

DNA methylation influences the differentiation of CD4^+^ T cells, thereby promoting the development of aGVHD. Significantly elevated levels of HMGB1 and STAT3 were detected in CD4^+^ T cells from patients with aGVHD, with HMGB1 expression positively correlated with STAT3 expression (144). More importantly, the expression levels of both proteins positively correlated with aGVHD severity. In further investigations, HMGB1 was shown to recruit DNA demethylases, including TET2, AID, and TDG, to form complexes in the *STAT3* promoter region (145). This process promotes hypomethylation of the *STAT3* promoter in CD4^+^ T cells from aGVHD patients, leading to abnormal STAT3 overexpression (Figure 3c). Previous studies have indicated that the balance between Tregs and Th17 cells is crucial for maintaining immune homeostasis in patients with aGVHD (146). During aGVHD progression, HMGB1 and STAT3, which are highly expressed in CD4^+^ T cells, promote Th17 lymphocyte induction while suppressing Treg differentiation. This imbalanced Treg/Th17 ratio ultimately triggers aGVHD.

However, most studies rely on PBMCs or whole tissues for global methylation analysis, and the inherent cellular heterogeneity of these samples limits the precise characterization of methylation patterns in specific cell subsets. Different cell subpopulations may exhibit markedly distinct methylation profiles, and their proportions or phenotypes may alter during immune responses. Although these studies have explored functional alterations in T cell subpopulations, they have not explicitly employed cell sorting or methylation quantification methods (such as single-cell sequencing) to distinguish methylation patterns among distinct cell subpopulations. These issues require further investigation and refinement in future research.

Although direct evidence regarding DNA methylation in heart transplantation remains relatively limited, existing studies suggest that methylation abnormalities can lead to vascular pathology in heart grafts. Given that vascular remodeling is a pathological hallmark of chronic heart graft dysfunction, future research should prioritize cell-type-specific methylation profiling of endothelial cells, vascular smooth muscle cells, and infiltrating immune cells in heart transplant recipients.

### 5.2 Histone methylation

**Figure 5:**
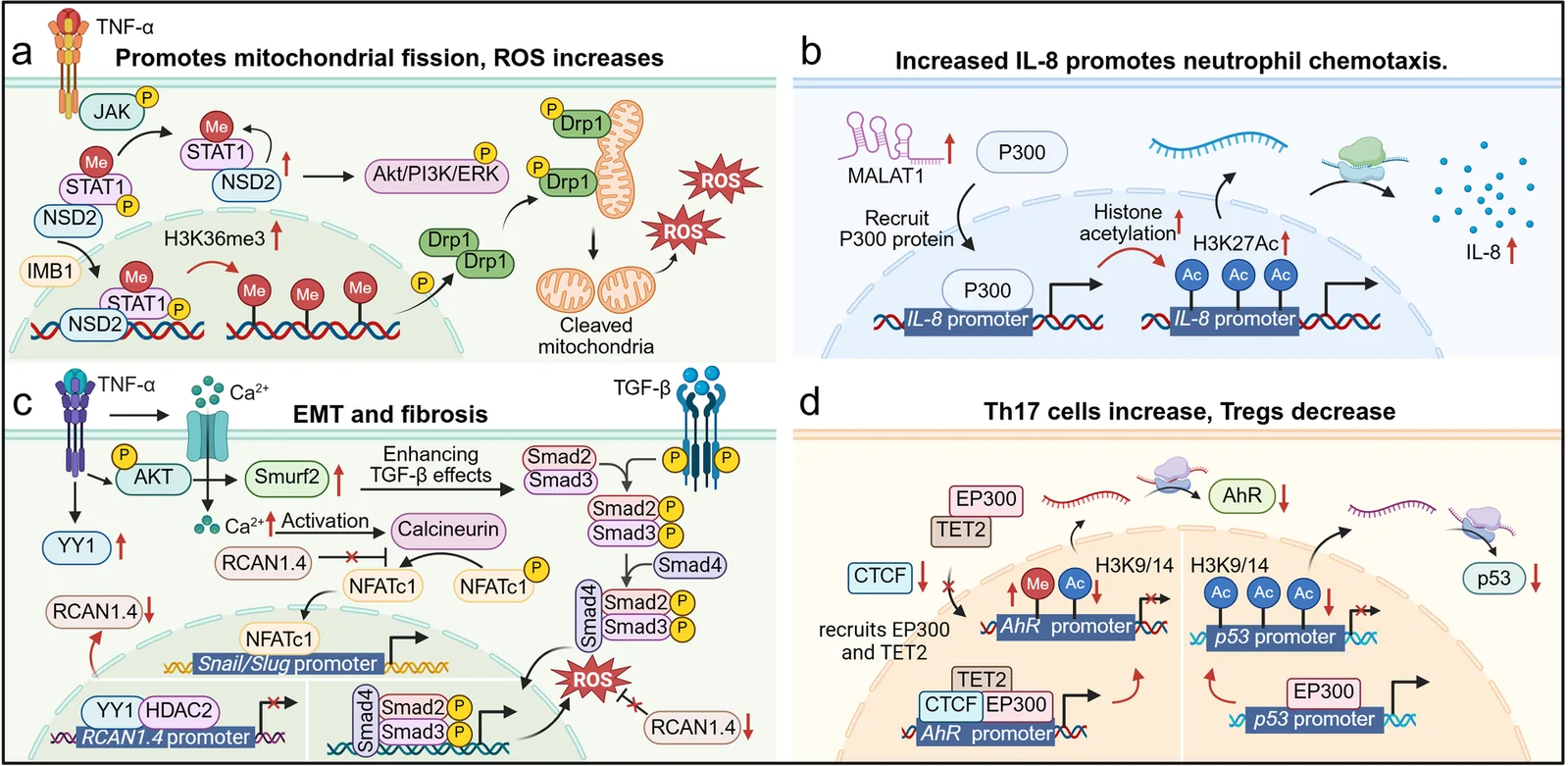
The role of histone modifications in transplant immunology. **(a)** Increased mitochondrial fission and reactive oxygen species (ROS) production. NSD2 interacts with STAT1 at K173 and induces STAT1 monomethylation, leading to its phosphorylation. This process ultimately exacerbates TNF-*α*-induced Drp-1-mediated mitochondrial fission, ROS accumulation, and fibrosis via the STAT1/ERK/PI3 K/Akt signaling pathway. **(b)** Increased IL-8 levels promote neutrophil chemotaxis. Increased lncRNA-MALAT1 expression leads to the recruitment of P300 to the nucleus, promoting H3K27 acetylation at the *IL-8* promoter and increasing IL-8 expression. **(c)** The epithelial‒mesenchymal transition and fibrosis. TNF-*α* signaling upregulates YY1 expression and induces the EMT via the Akt/Smurf2 signaling pathway. RCAN1.4 inhibits the TNF-*α*-induced EMT by regulating oxidative stress and the NFATc1 signaling pathway. However, upregulated YY1 binds to the HDAC2-containing *RCAN1.4* promoter region, suppressing RCAN1.4 expression and thereby promoting TNF-*α*-induced ROS production and EMT. **(d)** The number of Th17 cells increase, the number of Tregs decreases, and Th17/Treg imbalance occurs. CTCF recruits EP300 and TET2 to bind to the *AhR* promoter region. Low CTCF expression causes histone deacetylation and DNA hypermethylation at the *AhR* promoter, resulting in insufficient AhR expression in CD4^+^ T cells and reduced Treg differentiation. Furthermore, reduced CTCF results in low histone H3K9/K14 acetylation at the *p53* promoter and p53 downregulation, exacerbating the Th17/Treg imbalance.

Histone methylation precisely regulates DNA accessibility, thereby influencing the execution of immune cell functions (Figure 5).

#### 5.2.1 Acute rejection

Histone methylation influences AR through multiple pathways, including altering metabolism, interfering with T-cell activation, and inducing immune tolerance. Metabolomic analysis of kidney transplant samples from rats that developed acute rejection revealed increased H3K4me3 levels and decreased DNA methylation levels in PBMCs (147). Specifically, genes such as *Abcc4, Eif6*, and *Tbx1* are upregulated, while *Mef2d* is downregulated. These molecular changes enhance pathways such as *β*-oxidation, thereby driving an acute rejection response. In addition to influencing metabolism, histone methylation is intimately linked to AR. Research corroborated the pivotal role of EZH2 in transplant immunity (148). EZH2, the catalytic subunit of the Polycomb repressive complex 2 (PRC2), catalyzes H3K27me3 (149). Their study demonstrated that the administration of the EZH2 inhibitor DZNep after kidney transplantation effectively disrupted the activation and survival of allogeneic reactive T cells, leading to a marked improvement in AR outcomes and a reduction in histological damage and inflammatory cell infiltration in allogeneic kidney grafts. Modulating histone methyltransferases can further promote Treg differentiation. Among these, the SET domain-containing histone lysine methyltransferase 1 (SETDB1) plays a crucial role (37). As a principal H3K9 methyltransferase, SETDB1 profoundly influences transcriptional activity through the addition of methyl groups at H3K9 (150). The findings compellingly indicate that SETDB1 loss induces Treg differentiation by promoting specific histone methylation patterns. This differentiation process orchestrates substantial remodeling of the T-cell gene landscape by the activation of ATF transcription factors, thereby enhancing the immunosuppressive activity of T cells.

#### 5.2.2 Chronic rejection

Current research on histone methylation has only addressed its role in promoting mitochondrial fission in CR. Current research has revealed elevated levels of NSD2 and its product, H3K36me3, in individuals with chronic renal transplant rejection (151). Nuclear SET domain-containing protein 2 (NSD2), a histone methyltransferase that catalyzes lysine 36 methylation of histone H3 (152, 153), directly interacts with STAT1 at lysine K173. This interaction induces STAT1 monomethylation, which promotes STAT1 phosphorylation and IMB1-dependent nuclear translocation. This cascade subsequently leads to exacerbated Drp-1-mediated mitochondrial fission in TNF-*α*-induced epithelial cell models. IF/TA is driven by this process, which occurs through the STAT1/ERK/PI3 K/Akt signaling pathway (Figure 4a).

However, other studies on histone methylation related to CR are extremely rare. Future investigations are thus urgently needed to systematically delineate the repertoire of histone marks and associated methyltransferases that contribute to the maladaptive cellular responses underlying.

#### 5.2.3 Graft-vs.-host disease

The use of histone methylation inhibitors may influence GVHD through pathways such as regulating CD8^+^ T cells and promoting T-cell apoptosis. Research found that treatment with the histone methyltransferase inhibitor 3-deazabacitabine (DZNep) delayed the proliferation of donor-derived alloreactive CD8^+^ T cells and significantly reduced IL-2 production by these T cells *in vivo* (154). Furthermore, DZNep treatment markedly reduced the expression of IFN-*γ*, TNF-*α*, granzyme B, TRAIL, and Fas ligand in CD8^+^ T cells, suggesting its efficacy in suppressing effector T-cell activation and function. He et al. further validated the therapeutic efficacy of DZNep in GVHD treatment (155). Following the administration of DZNep *in vivo*, they successfully prevented ongoing GVHD in mice after allogeneic bone marrow transplantation. Mechanistic studies revealed that DZNep selectively reduces histone H3K27me3 levels, depletes the H3K27me3-specific histone methyltransferase Ezh2, and activates the proapoptotic gene Bim, which is normally suppressed in antigen-activated T cells. These findings consistently confirm that DZNep not only effectively controls GVHD but also preserves the efficacy of the graft against leukemic cells. Further research revealed that conditional Ezh2 gene knockout in donor T cells similarly and effectively suppressed GVHD in mice following allogeneic bone marrow transplantation during a one-year follow-up period (156). In the late stages of GVHD induction, Ezh2-deficient T cells exhibit impairments in sustained proliferation and expansion, and Ezh2 loss specifically impairs the differentiation of T cells into IFN-*γ*-producing effector cells. Notably, however, Ezh2 knockout did not affect the antileukemic activity of allogeneic reactive T cells, thereby improving recipient survival.

However, current understanding of the role of histone methylation in transplant immunity remains relatively limited. Most studies are based on pooled samples, making it difficult to avoid confounding factors related to cellular heterogeneity. The lack of single-cell resolution analyses and long-term follow-up data limits our in-depth understanding of the dynamic changes in histone methylation and their clinical significance.

### 5.3 Histone acetylation

The acetylation status of histones serves as a direct signal promoting gene expression, and its dynamic changes are crucial for the activation and differentiation of immune cells (Figure 5).

#### 5.3.1 Ischemia–reperfusion injury

Histone acetylation influences the process of fibrosis, Levine et al. reported that systemic HDAC inhibitors, including trichostatin (TSA) and the class I-specific inhibitor MS-275, ameliorated acute renal IRI and significantly reduced long-term fibrosis (157). Further research on HDAC inhibitors showed that the HDAC3-specific inhibitor RGFP966 mitigated cold-induced/transplant injury in a mouse kidney transplant model and markedly improved allogeneic kidney graft function (158). This inhibitor, along with *HDAC3* gene knockout, similarly protected rat proximal renal epithelial cells from cold-induced/rewarmed injury. Acetylation levels not only influence tissue injury and fibrosis but also affect tissue repair and regeneration. As early as 2008, Marumo et al. first demonstrated that histone acetylation levels in the proximal tubules of a mouse renal ischemia model are transiently reduced (159). During subsequent reperfusion, HDAC5 activity decreases, paralleling the recovery of histone acetylation. Studies have revealed that HDAC5 downregulation is closely associated with the acetylation levels and expression of bone morphogenetic protein 7 (BMP7), a key factor involved in renal repair and regeneration (160, 161).

#### 5.3.2 Chronic rejection

Recent investigations have illuminated the intricate roles of HDACs, particularly Sirtuin-1 and HDAC2, in modulating the immune responses and fibrotic processes that contribute to CRAD.

A compelling body of evidence implicates Sirt1 as a critical regulator of Treg function and a determinant of allograft survival. Research reported significantly reduced Sirt1 expression in rats with CRAD (162). Importantly, these investigators observed inverse correlations between Sirt1 expression levels and key indicators of renal dysfunction. These findings collectively suggest that Sirt1 may play a crucial role in the early stages of IF and inflammatory processes in CRAD. Moreover, Sirt1 is also associated with Tregs. In mouse models, pharmacological inhibition of Sirt1 significantly prolongs the survival time following allogeneic kidney transplantation (163). This protective effect is mechanistically linked to the known function of Sirt1 as a Class III HDAC that deacetylates Foxp3, thereby influencing Treg activity (164, 165). Indeed, studies have shown that Sirt1 deficiency leads to an increased presence of functional Tregs within allografts, a cellular phenotype associated with suppressed allogeneic kidney transplant rejection. Although both studies focused on the role of Sirt1 in kidney transplantation, their results presented apparent contradictions. This discrepancy does not represent a genuine contradiction but rather stems from differences in research models. It may be related to the pronounced cell type specificity, tissue specificity, and time point specificity of Sirt1's function. Its therapeutic application in kidney transplantation requires careful evaluation based on the specific pathological context and target mechanisms involved.

In parallel, emerging data suggest the involvement of HDAC2 in promoting proinflammatory responses and contributing to graft deterioration. Reduced HDAC2 expression was detected in CD8^+^ T cells and NKT-like lymphocytes following lung transplantation (166). This downregulation of HDAC2 in inflammatory immune infiltrates could lead to increased proinflammatory gene expression and a concomitant reduction in graft survival. Furthermore, a dysregulated molecular interaction involving HDAC2, YY1, and RCAN1.4 was found in the fibrotic lesions of chronic allogeneic kidney grafts in mice (167). It reported downregulated expression of RCAN1.4 and upregulated expression of YY1 in these fibrotic tissues. Mechanistically, HDAC2 was demonstrated that interacts with YY1 to form a multiprotein complex that represses RCAN1.4 transcription in response to TNF-*α*. RCAN1.4 has been shown to inhibit the TNF-*α*-induced EMT *in vitro* by modulating antioxidant stress and calmodulin/NFATc1 signaling (168, 169). These findings underscore the critical role of the HDAC2–YY1–RCAN1.4 axis in driving fibrotic remodeling and potentially contributing to CRAD.

#### 5.3.3 Graft-vs.-host disease

Histone acetylation exerts a multifaceted influence on the pathogenesis of aGVHD by orchestrating key cellular and molecular events, including the modulation of T cell activation, the promotion of pro-inflammatory cytokine release, and the disruption of the delicate Treg/Th17 balance. Research discovered that HDAC11 expression is significantly reduced in activated and effector T cells (170). Interestingly, HDAC11-deficient T cells exhibit increased proliferation and production of proinflammatory cytokines, suggesting that HDAC11 suppresses T-cell overactivation. Further studies found that HDAC11 binds to the promoter regions of *Eomes* and *Tbet* genes in quiescent T cells and dissociates upon T-cell activation. In HDAC11-knockout T cells, the expression levels of the *Eomes* and *Tbet* transcription factors increased, and accelerated aGVHD progression was observed in mouse models. Concurrently, Xu et al. focused on the role of SIRT1 in aGVHD (171). They discovered that SIRT1 expression is downregulated in CD4^+^ T cells from patients with aGVHD and that SIRT1 deficiency increases CD4^+^ T-cell activation. Researchers have further elucidated that the excessive activation of the IL-1*β* signaling pathway is the primary cause of SIRT1 deficiency in CD4^+^ T cells from aGVHD patients. This deficiency subsequently triggers the excessive activation of the PI3 K/Akt/mTOR pathway in CD4^+^ T cells, leading to the hyperacetylation and hyperphosphorylation of *STAT3*. This dysregulated signaling ultimately promotes excessive CD4^+^ T-cell activation and proinflammatory cytokine release, thereby inducing aGVHD.

Beyond its established influence on T cell activation, histone acetylation is a critical determinant of CD4^+^ T cell lineage commitment and terminal differentiation. An imbalance in the Treg/Th17 cell ratio accelerates the progression of aGVHD, with the aryl hydrocarbon receptor (AhR) playing a key regulatory role in the differentiation of both Treg and Th17 cells (172, 173). Building on this foundation, research investigated the specific role of AhR in aGVHD pathogenesis (174). It revealed significantly downregulated AhR expression in CD4^+^ T cells from aGVHD patients. And further progress elucidated the molecular mechanisms governing AhR expression and discovered that the CTCF protein recruits EP300 and TET2 to the *AhR* promoter region. By increasing the acetylation of histone H3K9/K14 and DNA demethylation at this locus, CTCF promotes AhR expression. Crucially, they previously demonstrated that CTCF expression is universally reduced in CD4^+^ T cells from aGVHD patients (175). This reduction in CTCF expression leads to the hypoacetylation of histones and the hypermethylation of DNA in the *AhR* promoter region, consequently resulting in insufficient AhR expression in CD4^+^ T cells from patients with aGVHD. Complementing these findings, low CTCF expression was found to be associated with reduced histone H3K9/K14 acetylation at the *p53* promoter and consequently downregulated p53 expression. Ultimately, the dysregulation of these molecular mechanisms contributes to the aberrant Treg/Th17 balance that mediates the development of aGVHD.

Although progress has been made in the study of histone acetylation, it faces challenges related to cellular heterogeneity, making it difficult to distinguish specific modification patterns among different immune cell subsets in whole-sample analyses.

Compared with DNA methylation and non-coding RNAs, studies investigating histone modifications in heart transplantation remain relatively limited. Nevertheless, accumulating evidence indicates that histone-modifying enzymes regulate key biological processes closely associated with cardiac graft outcomes, including T-cell activation, Treg stability, inflammatory responses, fibrosis, and endothelial dysfunction. Given that ischemia–reperfusion injury and cardiac allograft vasculopathy represent the principal causes of early and late graft injury after heart transplantation, further studies are warranted to define the histone modification landscape in these cardiac-specific pathological settings.

### 5.4 MicroRNAs

**Figure 6:**
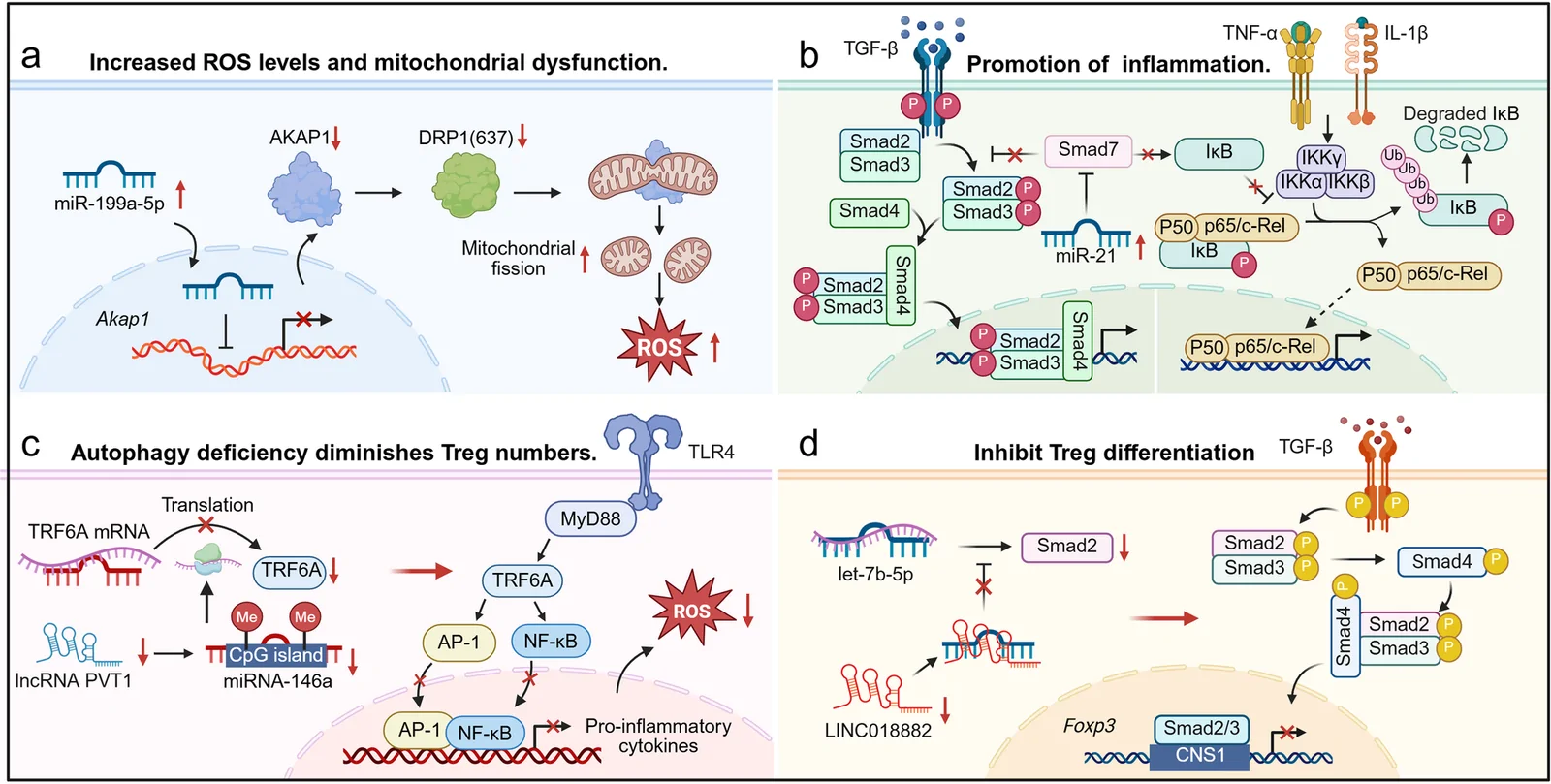
The role of non-coding RNA in transplant immunology. **(a)** Increased reactive oxygen species (ROS) levels and mitochondrial dysfunction. In ischemia‒reperfusion injury, elevated miR-199a-5p expression suppresses AKAP1 expression, thereby reducing the phosphorylation of DRP1 at amino acid 637. This process promotes mitochondrial lysis, generating ROS and leading to mitochondrial dysfunction. **(b)** Promotion of cellular inflammatory responses. miR-21 downregulates Smad7, which inhibits Smad3, thereby promoting TGF-*β*-mediated fibrosis and injury. Concurrently, it increases NF-*κ*B-driven inflammation through Smad7-dependent mechanisms. **(c)** The inhibition of Treg autophagy reduces the number of Tregs. lncRNA-PVT1 directly targets and suppresses miR-146a expression, thereby promoting TRAF6 expression. Significant downregulation of lncRNA-PVT1 expression leads to reduced TRAF6 expression, inhibits Treg cell autophagy, and enhances rejection responses. **(d)** Treg differentiation is suppressed. The lncRNA LINC01882 competitively binds to miRNA-let-7b-5p, thereby protecting its downstream molecule SMAD2 from degradation. Downregulation of the lncRNA LINC01882 leads to reduced SMAD2 expression, subsequent inhibition of the SMAD2 signaling pathway, and decreased Treg differentiation.

miRNAs precisely regulate cellular communication and responses by binding to target mRNA molecules, thereby silencing or degrading the synthesis of target proteins (Figure 6).

#### 5.4.1 Ischemia–reperfusion injury

Multiple studies have revealed the differential roles of several miRNAs in various tissue IRI models, ranging from promoting apoptosis and disrupting mitochondrial dynamics to enhancing cellular viability and inhibiting inflammatory signaling pathways. Shi et al. identified increased miR-199a-5p expression in a renal IRI model and observed its direct targeting of anchoring protein 1 (AKAP1). This promotes mitochondrial fission by activating the phosphorylation of dynamin-related protein 1 (Drp1) at position S637 (176). Conversely, AKAP1 overexpression maintained Drp1-S637 phosphorylation and reduced mitochondrial fragmentation (Figure 6a). Moreover, mitochondrial dysfunction was alleviated upon application of the miR-199a-5p inhibitor. The findings indicate that miR-199a-5p activation reduces AKAP1 expression, disrupts mitochondrial dynamics, and ultimately exacerbates renal IRI.

In contrast, significantly decreased miR-449b-5p expression and markedly increased HMGB1 expression were found in a liver IRI model (177). This revealed a direct interaction between miR-449b-5p and the 3′ untranslated region of the HMGB1 mRNA. The regulation of miR-449b-5p overexpression markedly increased cell viability and inhibited apoptosis in H/R-exposed L02 cells. Furthermore, miR-449b-5p significantly reduced ALT and AST levels, highlighting its protective role through inhibition of the HMGB1/NF-*κ*B pathway. Previously, we discussed how HMGB1 regulates its expression in CD4^+^ T cells by modulating the DNA methylation levels of STAT3. Could miR-449b-5p also influence subsequent immune responses by regulating DNA methylation levels in CD4^+^ T cells through its modulation of HMGB1? This question warrants further investigation in future studies. Another investigation revealed that hepatic miR-128-3p levels negatively correlated with Rnd3 expression, with the Rnd3 mRNA serving as a direct target of miR-128-3p (178). The level of Rnd3 mRNA increased as the reperfusion time extended, and finally peaked after 6 h of reperfusion. Knockdown of miR-128-3p led to the upregulation of Rnd3 mRNA and protein levels, subsequently inhibiting the NF-*κ*B pathway by downregulating NF-*κ*B p65. This regulator*y* axis ultimately reduced the serum levels of inflammatory factors and AST/ALT, which are associated with NF-*κ*B activation, indicating that inhibiting miR-128-3p protects against hepatic IRI. Intriguingly, miR-128-3p expression progressively declined post-reperfusion following 1 h of ischemia, while Rnd3 mRNA levels rose with extended reperfusion. Why does this protective mechanism occur in IRI? Could it potentially exert its effects by influencing other mechanisms? The protective role of this mechanism in IRI and its potential to influence other pathways require further investigation.

#### 5.4.2 Acute rejection

miRNAs can influence AR by regulating CD4^+^ T cells, inhibiting apoptosis, and modulating cytokines. For instance, in the context of acute heart allograft rejection, a significant reduction in the protein expression of nuclear receptor 4A1 (Nr4A1), also known as Nur77 was detected (179). It revealed that the microRNA let-7a directly binds to the 3′UTR of the NR4A1 mRNA, thereby decreasing Nr4A1 protein levels through posttranscriptional regulation. Conversely, the inhibition of let-7a expression demonstrably upregulated Nr4A1 protein expression. The previous investigations have established Nr4A1 as a critical regulator that targets CD4^+^ T cells to attenuate allogeneic graft rejection (180). Collectively, these findings suggest that let-7a-mediated suppression of the Nr4A1 protein may exacerbate acute rejection following heart transplantation by decreasing the protective effects of Nr4A1 on CD4^+^ T-cell populations. MiRNAs are also implicated in apoptosis, the downregulation of miR-10b was a prominent molecular hallmark of human kidney allograft rejection, while transfection with a miR-10b mimic produced a protective effect (181). miR-10b downregulation induced glomerular endothelial cell apoptosis by targeting the key pro-apoptotic protein BCL2L11 and promoted the release of pro-inflammatory cytokines and chemokines, facilitating macrophage chemotaxis, thereby exacerbating renal inflammation and rejection. This study highlights the critical protective role of miR-10b in preserving glomerular endothelial cell survival and suppressing proinflammatory signaling pathways.

As for cytokines, miR-505-5p expression significantly reduced in liver transplant recipients experiencing AR (182). Experimental overexpression of miR-505-5p effectively attenuated the release of proinflammatory cytokines both *in vitro* and *in vivo*, a function attributed to its inhibition of the NF-*κ*B and MAPK signaling cascades. Crucially, miR-505-5p promoted macrophage M2 polarization via the Myd88/TRAF6 axis, thereby enhancing immunosuppression and tissue repair (183). The effects of miRNAs on cytokines were also observed in another mouse heart transplantation model. The significant upregulation of miR-669b-3p exerts its effects through the negative regulation of indoleamine 2,3-dioxygenase (IDO) (184). Furthermore, miR-669b-3p modulates the expression of inflammatory cytokines, including TNF-*α* and IL-10, thereby promoting a shift toward a Th2-dominant immune response. Furthermore, the application of miR-669b-3p inhibitors significantly reduces the T cell proliferation stimulation index. This modulation of the immune milieu may contribute to the amelioration of acute rejection in heart transplant recipients.

#### 5.4.3 Chronic rejection

miRNAs can primarily regulate T-cell immune responses (particularly Th1/Th17), thereby influencing CR. Zhang et al. reported upregulated expression of miR-155, which is essential for Th17 differentiation and survival, in individuals with chronic heart transplant rejection, with miR-155 knockout improving outcomes by limiting Th1/Th17 immunity (185). miRNAs also influence fibrosis in CR. miR-21-5p was identified among six differentially expressed miRNAs in kidney transplant recipients with CAD (186); these results are consistent with Ben-Dov et al.'s report of elevated miR-21 expression in IF/TA kidneys (187). miR-21 strongly promotes renal fibrosis by downregulating Smad7, thus disinhibiting Smad3 and amplifying NF-*κ*B-driven inflammation via Smad7-dependent mechanisms (188–190) (Figure 6b). Beyond renal pathology, miR-21 critically modulates macrophage behavior. The pronounced expression of miR-21 was detected in macrophages during chronic allograft vasculopathy (CAV) after heart transplantation (191). Crucially, the conditional deletion of miR-21 in macrophages, prolonged allograft survival. Targeting miR-21 to reprogram macrophage metabolism delayed CAV. Other miRNAs also play important roles. In lung transplant recipients, Xu et al. investigated patient PBMCs using the Taqman Low-Density miRNA Array method and found that DSA-induced immune responses primarily affected miRNAs involved in TGF-*β* and B-cell receptor signaling pathways (192). However, these analyses were restricted to bioinformatic profiling of miRNA expression, lacking specific functional validation. This absence of direct experimentation significantly limits the inferred functional relevance of the proposed relationships between specific miRNAs and TGF-*β* or B-cell receptor signaling pathways. Another study on BOS found elevated levels of miR-144 expression in patients, inversely correlating with the expression of TGIF1 (a Smad co-inhibitor) (193). miR-144 overexpression reduces TGIF1 expression and increases the expression of SMAD2/4, FGF6, TGF-*β*, and VEGF, thus promoting fibrosis in individuals with BOS. Conversely, knocking down miR-144 diminishes fibrogenesis in pulmonary fibroblasts.

#### 5.4.4 Graft-vs.-host disease

miRNAs modulate innate immune cell populations and T cell effector functions, collectively influencing the development and severity of GVHD. Studies of aGVHD revealed markedly elevated levels of miR-29a in patient serum (194). Mechanistic investigations demonstrated that miR-29a induces dendritic cell maturation and activation through the TLR7/8 signaling pathway, subsequently activating the NF-*κ*B pathway to promote the secretion of the proinflammatory cytokines TNF-*α* and IL-6. Treatment with LNA anti miR-29a significantly improved survival in a mouse model of aGVHD while retaining GVL effects, unveiling a novel therapeutic target in aGVHD treatment or prevention. Additionally, other miRNAs can regulate T cells. Wu et al. reported that the miR-17-92 cluster is a key regulator that promotes CD4^+^ T-cell activation, proliferation, survival, and Th1 differentiation while simultaneously inhibiting Th2 and iTreg differentiation (195). Researchers have employed LNA antagonists to block the miR-17-92 cluster and found that this blockade significantly suppressed the expansion of allogeneic reactive T cells and IFN-*γ* production, effectively prolonging survival in GVHD recipients while preserving GVL effects.

Furthermore, Wu et al. reported a significant upregulation of miR-31 expression in allogeneic T cells (196). In these cells, miR-31 promotes a hypoxic adaptation by inhibiting FIH1, thereby increasing HIF1*α* signaling and glucose metabolism. These metabolic and signaling alterations further promote Th17 cell differentiation while suppressing Treg cell generation and function, exacerbating the immune imbalance in individuals with cGVHD. This study confirms that LNA anti-microRNA targeting miR-31 is effective not only in gene knockout models but also in alleviating cGVHD following systemic administration. Its efficacy persists even with delayed treatment, thereby enhancing its potential for clinical translation. These studies demonstrated that application of LNAs to antagonise the corresponding miRNAs exerts an anti-GVHD effect while preserving the GVL effect. However, the potential for clinical translation of therapeutic efficacy requires more rigorous validation, particularly in terms of safety, human efficacy, and drug delivery.

Current research on miRNAs spans a wide range of areas, from mitochondrial dynamics and parenchymal cell proliferation to T cell activation. As the most extensively studied RNA within the ncRNA category, miRNAs can regulate DNA methylation and histone modifications. However, studies integrating these interdependent regulatory mechanisms within transplant immunology remain scarce. Moreover, these studies primarily rely on mouse models and lack sufficient human sample data. Although research may reference human contexts, there remains a significant gap in validating the significance of identified targets across large human patient cohorts and correlating them with clinical outcomes—such as disease severity, recurrence rates, and survival rates.

Collectively, heart transplantation studies have identified several cardiac-specific miRNAs, including let-7a, miR-669b-3p and miR-155, that regulate myocardial immune responses, T-cell activation and cardiac allograft vasculopathy. These observations suggest that miRNAs currently represent the most mature epigenetic biomarkers and therapeutic candidates in cardiac transplantation.

### 5.5 LncRNAs

LncRNAs influence transcriptional regulation and cellular signaling pathways through multiple mechanisms (Figure 6).

#### 5.5.1 Ischemia–reperfusion injury

LncRNAs play intricate roles in cellular reprogramming, metabolic regulation, and inflammatory signaling, positioning them as pivotal elements for understanding and treating IRI. In renal IRI, researchers discovered that small extracellular vesicles (IRI-sEVs) deliver the lncRNA WAC-AS1, which can reprogram glucose metabolism in adjacent tubular epithelial cells (197). LncRNA WAC-AS1, functioning as a competitive endogenous RNA, has been found to promote GFPT1 expression by directly binding to miR-449b-5p, thereby promoting HBP metabolic reprogramming. LncRNA-WAC-AS1 fosters HBP metabolic acceleration and BACH2 O-GlcNAcylation in neighboring and distant cell populations and thus potentiates the transcriptional suppression of SLC7A11 and GPX4 induced by BACH2, facilitating wide propagation of the “wave of ferroptosis”. The wide propagation of the “wave of ferroptosis” provokes extensive tubular necrosis, thus aggravating IRI-induced renal injury and DGF during transplantation to a large extent.

LncRNAs can also influence the IRI process by regulating histone modifications, elevated expression of both IL-8 and MALAT1 has been observed in lung tissue (198). Further studies revealed that MALAT1 interacts with the p300 protein, increasing H3K27ac levels at the *IL-8* promoter. This study confirmed that silencing MALAT1 and downregulating its interaction with p300 reduces IL-8 expression, thereby inhibiting neutrophil chemotaxis and significantly mitigating inflammatory injury after lung transplantation ischemia‒reperfusion.

#### 5.5.2 Acute rejection

LncRNAs play a pivotal role in the immunoregulatory network involved in AR, ranging from modulating Th1 cell activation to promoting Treg autophagy. Focusing on an acute cardiac rejection model, upregulation of lncRNA-A930015D03Rik and mouse lncRNA 1055 was observed (199). By inhibiting lncRNA-A930015D03Rik and mouselincRNA1055, the researchers observed the suppression of IL-12R*β*1 and IFN-*γ* expression in Th1 cells, suggesting that these lncRNAs may influence rejection processes by regulating Th1 cell activation and function. In addition to influencing Th1 cells, lncRNAs can also affect Tregs. During heart transplant rejection, the expression of lncPVT1 in Tregs is significantly downregulated and negatively correlated with the expression of miR-146a (200). PVT1 overexpression directly targeted miR-146a, thereby upregulating TNF receptor-associated factor (TRAF) 6 expression, promoting autophagy in Tregs, and synergistically attenuating rejection (Figure 6c). Notably, miR-146a overexpression reversed PVT1-induced Treg autophagy and suppressed PVT1-mediated TRAF6 expression, suggesting the importance of the PVT1–miR-146a–TRAF6 axis in regulating Treg function. Furthermore, in cases of kidney transplant rejection, the expression of lncRNA-ATB was significantly elevated, while that of miRNA-200c was reduced. lncRNA-ATB could function as a ceRNA by competitively biding common microRNAs, especially miR-200c which can repress TGF-*β*-mediated EMT and invasion (201). The study indicates that lncRNA-ATB not only influences renal cell phenotype but also modulates the nephrotoxicity of CsA.

#### 5.5.3 Graft-vs.-host disease

Among lncRNAs, distinct populations exhibit opposing roles in aGvHD; some confer protection, whereas others actively promote disease progression. Research detected significantly downregulated expression of the lncRNA LINC01882 in the peripheral blood of patients with aGVHD (202). In-depth mechanistic investigations revealed that LINC01882 competitively binds to the microRNA let-7b-5p, thereby protecting its downstream target gene smad2 from degradation. Since *Smad2* is a key promoter of Treg differentiation, the overexpression of LINC01882 promotes Treg differentiation and significantly ameliorates aGVHD (Figure 6d). In contrast, certain lncRNAs promote aGVHD. Among these, lnc-AC145676.2.1–6-3 and STX3 were upregulated in the aGVHD mouse model (203). Lnc-AC145676.2.1–6-3 influences disease progression by regulating the downstream hsa-miR-1292-3p/STX3 axis. STX3 subsequently promotes intestinal aGVHD development by increasing autophagy levels through the PTEN/AKT/mTOR pathway. In another study, previous researchers confirmed the upregulation of lnc-AC145676.2.1-6-3 in peripheral blood mononuclear cells (PBMCs) from patients with intestinal aGVHD, where this lncRNA influences intestinal aGVHD progression by regulating the hsa-miR-3064-5p/IL-1*β* axis (204). However, both studies primarily relied on bioinformatics analysis without sufficient functional validation. There was a lack of direct *in vivo* experiments involving siRNA knockdown or mimic overexpression of lnc-AC145676.2.1-6 in aGVHD mouse models. This significantly undermines the credibility of elucidating its effect pathways.

In these studies, we found that lncRNAs primarily exert regulatory effects through interactions with miRNAs, epigenetic modification enzymes, and other factors. This highlights the interplay of epigenetics within transplant immunology. However, many studies rely solely on bioinformatics analysis and *in vitro* cell experiments, raising doubts about the validity of their conclusions.

### 5.6 CircRNAs

CircRNAs have emerged as novel regulatory elements, functioning by sponge-like adsorption of miRNAs or serving as scaffolds for transcription factors.

#### 5.6.1 Ischemia–reperfusion injury

Circular RNAs play increasingly prominent roles in regulating cell fate and tissue injury. Studies have indicated that specific circRNAs are crucial regulators of cardiac and hepatic IRI, influencing cell death patterns and metabolic homeostasis. In a rat heart transplantation model, the expression of circFoxo3, miR-433, and miR-136 was significantly upregulated in IRI hearts and H/R-injured cardiomyocytes (205). Further investigations revealed that ROS increase circFoxo3 expression in cardiomyocytes. Functioning as a “molecular sponge”, circFoxo3 binds multiple miRNAs, including miR-433 and miR-136 (206). Both miR-433 and miR-136 have been established as pathogenic factors involved in cell death (207, 208). Therefore, siRNA-mediated knockdown of circFoxo3 effectively reduced H/R-induced cardiomyocyte apoptosis and death while mitigating mitochondrial damage *in vitro*. These findings suggest that circFoxo3 may amplify the pro-apoptotic effects of miR-433 and miR-136 through its sponge activity, thereby exacerbating myocardial IRI.

Currently, research on the mechanisms of action of circRNAs in the field of transplant immunology remains in its infancy. The field continues to face numerous challenges, including limitations such as insufficient depth and breadth of research, significant cellular heterogeneity, and a lack of validation through single-cell, spatially resolved, and large-scale longitudinal clinical data, as well as delays in translational research. These constraints hinder our comprehensive understanding of the potential functions of circRNAs and impede their clinical translation as novel biomarkers or therapeutic targets.

## 6 Applications of epigenetics in transplantation

Epigenetics offers transformative potential in transplantation, serving as both a non-invasive biomarker for early rejection detection and a target for inducing immune tolerance. By directly intervening in epigenetic mechanisms, this approach could drastically diminish reliance on current immunosuppressive agents (Table 1).

**Table 1:** Applications of epigenetics in transplantation.

Type of rejection	Transplant type	Epigenetic modification	Target	Source	Species	Application	Function	References
Acute rejection	Kidney transplantation	Histone methylation	EZH2	CD4^+^/CD8^+^ T cell	Rat	Inhibitor	Ameliorate	(148)
		Histone acetylation	Sirt-1	CD4^+^ T cell	Mice	Inhibitor	Suppress	(163)
		miRNA	miRNA-142-5p,miRNA-155	PBMC	Human	Biomarker	Diagnose	(209)
			miRNA-150-5p	PBMC	Human	Biomarker	Diagnose	(214)
			miRNA-99a	Serum	Human	Biomarker	Detect	(215)
		lncRNA	lncRNA-ATP1A1-AS1	—	Human	Biomarker	Predict	(218)
			lncRNA-AF264622	Peripheral blood	Human	Biomarker	Predict	(223)
		circRNA	circ-0001334	Urine	Human	Biomarker	Predict	(221)
	Heart Transplantation	DNA methylation	Foxp3	Treg	Mice	Antagonist	Alleviate	(233)
			—	Treg, CD8^+^ T cell	Mice	Cytokine, Antagonist	Suppress	(234)
		histone acetylation	Foxp3	Treg	Mice	Cytokine	Suppress	(36)
		miRNA	miRNA-10a,miRNA-31	Tissue, Serum	Human	Biomarker	Diagnose	(33)
			miRNA-144-3p,miRNA-652-3p	Serum	Human	Biomarker	Diagnose	(212)
			FOXO1	CD4^+^ T cell	Mice	miRNA, Inhibitor	Suppress	(237)
		lncRNA	miRNA-146a	Treg	Mice	lncRNA	Suppress	(200)
			NLRP3	DC	Mice	lncRNA	Suppress	(228)
			DC-SIGN	DC	Mice	lncRNA	Prevent	(238)
		circRNA	circ-FSCN1	DC	Mice	—	Prevent	(229)
	Liver Transplantation	miRNA	miRNA-199a-3p	Plasma	Rat	Biomarker	Predict	(217)
			miRNA-301a	Tissue	Rat	Biomarker	Diagnose	(222)
Chronic rejection	Kidney Transplantation	miRNA	miRNA-142-5p	PBMC	Human	Biomarker	Predict	(210)
		lncRNA	lncRNA-AC093673.5	—	Human	Biomarker	Predict	(219)
	Lung Transplantation	Histone methylation	EZH2	—	Mice	Inhibitor	Suppress	(242)
		Histone acetylation	HDAC2	CD8^+^ T, NKT-like	Human	—	Treat	(166)
	Heart Transplantation	circRNA	circRNA-MAP2K2	DC	Mice	—	Prevent	(230)
			circRNA-Malat1	DC, Treg	Mice	Cytokine	Prevent	(231)
Graft vs. host disease	Hematopoietic stem cell transplantation	DNA methylation	*Foxp3*	iTreg	Mice	Vitamin C	Prevent	(13, 225)
			*iCaspase-9*	—	Human	Antagonist	Treat	(232)
			DNMT1	NK cell	Mice	RNA	Alleviate	(241)
		Histone methylation	EZH2	CD8^+^ T cell	Mice	Inhibitor	Suppress	(146)
		Histone acetylation	BRD	DC, T cell	Mice	Inhibitor	Suppress	(34)
			Sirt-1	CD4^+^ T cell	Mice	Inhibitor	Suppress	(224)
			HDAC	—	Mice	Inhibitor	Reduce	(235)
			—	CD326+IECs	Mice	SCFAs	Suppress	(236)
			IL-1R, IL-6R, *FOXP3*	nTreg	Human	Vitamin A	Suppress	(240)
		miRNA	miRNA-17-92	CD4^+^ T cell	Mice	RNA	Suppress	(195)
			miRNA-423, miRNA-199a-3p	Plasma	Human	Biomarker	Predict	(211)
			miRNA-455-3p, miRNA-5787	Plasma	Human	Biomarker	Diagnose	(213)
			miRNA-365-3p, miRNA-148-3p	Serum	Human	Biomarker	Predict	(216)
			TRAF6	tTreg	Human	miRNA	Suppress	(226)
			*FOXP3*	Treg	Human	miRNA	Suppress	(227)
			AKT3, BIM	CD4^+^ T cell	Human	miRNA	Treat	(239)
		lncRNA	lnc-MAF4, IFNG-AS1	PBMC	Human	Biomarker	Diagnose	(220)

### 6.1 Epigenetic biomarkers

Epigenetic biomarkers (primarily miRNAs and lncRNAs) play crucial roles in the early prediction, diagnosis, and determination of the prognosis of transplant rejection. We shall first discuss the application of miRNAs as biomarkers. For AR, miR-142-5p and miR-155 expression was increased in PBMCs of kidney transplant recipients compared to healthy controls in a clinical cohort (209). Further biopsy tissue analysis indicated that levels of miR-142-5p and miR-155 within the graft, particularly miR-142-5p (AUC ≈ 0.99), could highly accurately identify AR. Moreover, their study revealed a predictive association between these miRNAs and graft function (eGFR). For CR, miR-142-5p was overexpressed in PBMCs from kidney transplant recipients experiencing CAMR, a finding validated in an independent cohort (210). miR-142-5p demonstrated good diagnostic performance with a ROC AUC of 0.74 in distinguishing AMR from stable graft function. Regarding GVHD, extensive human sample validation revealed that the expression of miR-423, miR-199a-3p, miR-93, and miR-377 was significantly upregulated in patients with aGVHD (211). A predictive model based on these four miRNAs demonstrated robust aGVHD probability prediction capabilities, with AUC values ranging from 0.76 to 0.85, enabling detection prior to aGVHD onset. Additional studies have indicated that miRNAs may serve as potential biomarkers for predicting or diagnosing rejection reactions. For instance, miR-144-3p and miR-652-3p predict acute cellular rejection in cardiac transplant recipients; miR-150-5p is used for diagnosing renal transplant rejection; miR-455-3p, miR-5787, and miR-548a-3p are markers of an aGVHD diagnosis and so on (212–217).

Although these studies demonstrate promising diagnostic performance, like many studies in this field, they typically rely on cross-sectional data or short-term follow-up, which limits the ability to establish robust longitudinal clinical correlations—a factor that is critical for sustained prognostic utility. Furthermore, variability in PBMC composition across different patients and time points may confound overall miRNA expression analysis, highlighting the need for cell-type-specific profiling.

Next, we shall discuss the application of lncRNAs as biomarkers. For AR, Zhang et al. employed univariate and multivariate Cox regression analyses alongside internal validation using training/testing sets to select four optimal lncRNAs—ATP1A1-AS1, CTD-3080P12.3, EMX2OS, and LINC00645—and construct a 4-lncRNA risk scoring model (218). The model demonstrated good sensitivity and specificity in predicting graft survival at 1, 2, and 3 years post-biopsy, with AUC values of 0.891, 0.836, and 0.733, respectively. Logistic regression and ROC analyses further confirmed the significant association of these four lncRNAs with AR. For CR, researchers constructed a six-lncRNA signature model including AC126763.1 from the dataset, achieving an AUC of 0.94 for predicting chronic injury and 0.86 for predicting progressive injury (219). Furthermore, they identified lncRNA-AC093673.5 as the only lncRNA significantly correlated with all three endpoints (chronic injury, progressive injury, graft failure) with AUC values of 0.64, 0.77, and 0.69, respectively. Both studies were retrospective analyses of existing clinical cohort data conducted to validate the research findings; however, no new samples were collected or processed. The impact of sample processing on reproducibility cannot be directly assessed, as reproducibility depends on the quality of original studies and data accessibility. Regarding GVHD, the Jafari team observed significantly higher expression levels of IFNG-AS1 and MAF4 in the aGVHD group compared to the non-aGVHD group at 28 days post-transplant and on the final day (IFNG-AS1: *p* = 0.0328, *p* = 0.0039; MAF4: *p* < 0.0001) in a small clinical cohort study (220). ROC analysis indicated that IFNG-AS1 and MAF4 effectively distinguished aGVHD from non-aGVHD patients 28 days post-transplant and beyond, demonstrating high sensitivity and specificity. For instance, at 28 days, MAF4 exhibited 84% specificity and 84.62% sensitivity; IFNG-AS1 showed 76% specificity and 76.92% sensitivity. By measuring expression at multiple time points, this study observed dynamic changes in lncRNA expression, aiding in assessing their relevance to disease progression. However, the sample size was small and external validation was not performed. Further studies elucidate the application of lncRNAs in identifying biomarkers for transplant rejection (221–223). Although these studies provide valuable insights into potential lncRNA biomarkers, a common challenge is the reliance on whole-tissue or PBMC analyses. This approach is susceptible to confounding effects from cellular heterogeneity, making it difficult to determine the precise cellular origin of epigenetic signals. Future studies will need to be validated in larger, prospective cohorts with robust longitudinal follow-up and, ideally, should incorporate cell sorting or single-cell approaches to confirm cell-type-specific associations and clinical trajectories.

Through non-invasive detection of these epigenetic markers (e.g., via peripheral blood), clinicians can identify potential rejection responses earlier, adjust immunosuppression regimens in a timely manner, avoid overtreatment or undertreatment, ultimately improving transplant success rates and enhancing long-term outcomes for recipients. However, translating epigenetic biomarkers into routine clinical practice requires larger-scale, multicenter, and longitudinal studies to rigorously address the issue of cellular heterogeneity, standardize sample collection and processing, and establish clear associations with long-term clinical outcomes.

### 6.2 Transplantation tolerance

**Figure 7:**
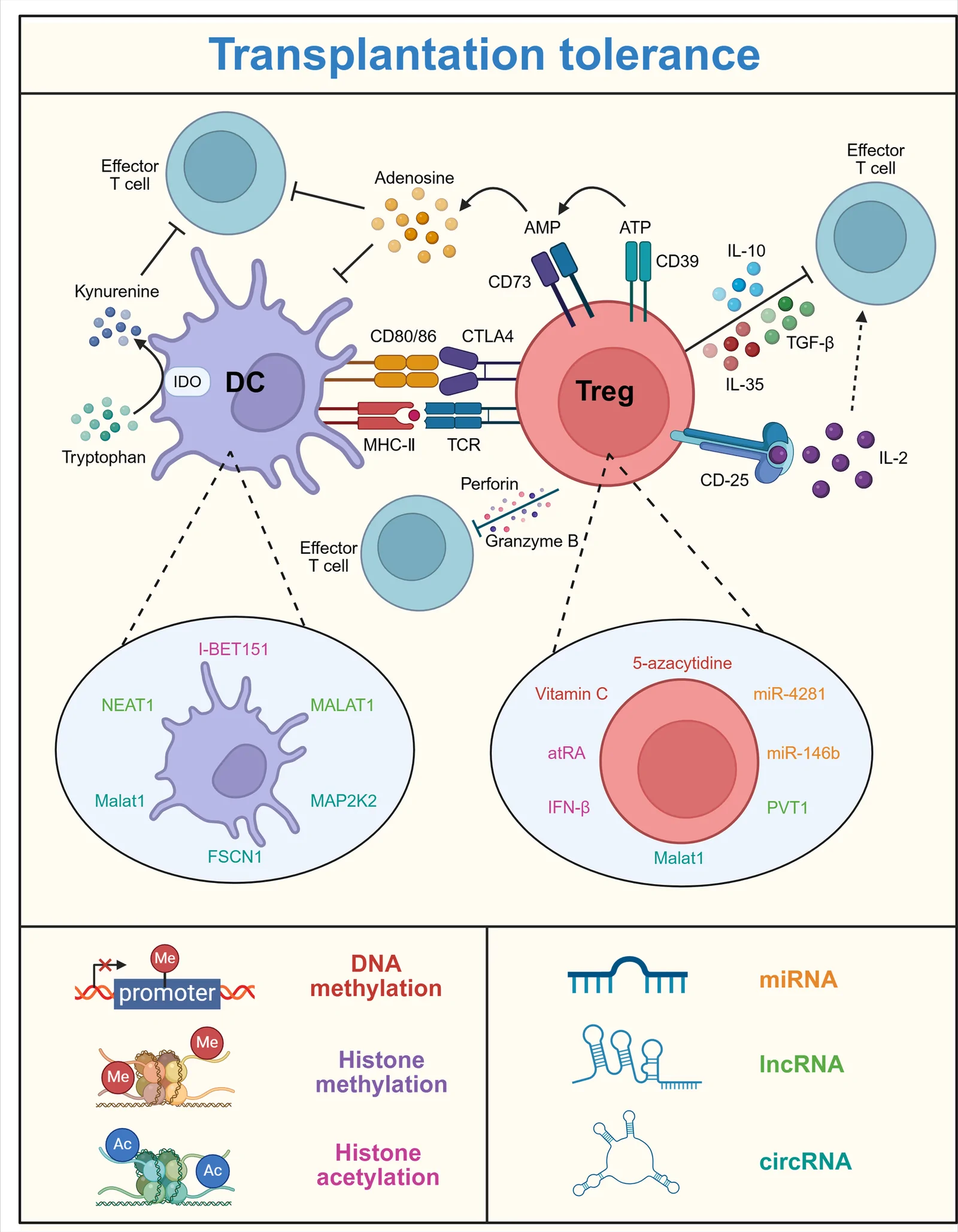
Transplantation tolerance and epigenetic regulation. This diagram illustrates the fundamental processes of immune tolerance and the epigenetic regulation of tolerance induction by DCs and Tregs. Peripheral transplant tolerance is mediated primarily by Tregs and DCs: Tregs suppress or kill effector T cells through mechanisms such as the secretion of cytokines like IL-10, the promotion of ATP conversion to adenosine, the depletion of IL-2, and the secretion of perforin and granzyme B. DCs promote kynurenine production to suppress effector T cells while also being inhibited by adenosine. The epigenetic regulation of Tregs and DCs induces transplant tolerance. Epigenetic mechanisms include DNA methylation, histone modifications, and non-coding RNAs, with different font colors representing distinct epigenetic regulatory pathways.

Transplant rejection is a manifestation of immune dysregulation and is mitigated by robust immune tolerance mechanisms. Central tolerance, mediated by thymic and bone marrow selection processes, eliminates autoreactive T- and B-cell clones recognizing endogenous antigens, thereby establishing self-identity. Nonetheless, self-reactive lymphocytes can escape central surveillance and infiltrate peripheral tissues. Here, peripheral tolerance is enforced by a complex interplay of immunosuppressive cellular effectors, including Tregs, that actively dampen immune responses via direct contact and cytokine secretion (e.g., IL-10 and TGF-*β*). Immune-privileged sites, which are characterized by anatomical barriers and local immunosuppressive microenvironments, further limit immune attack. Moreover, the differentiation and functional programming of APCs, particularly DCs, are critical for dictating immune outcomes toward tolerance or activation. Therefore, epigenetic mechanisms can be utilized to regulate Tregs, DCs, and other immune cells to suppress rejection responses and induce peripheral immune tolerance (Figure 7).

In the previous sections, we examined several epigenetic mechanisms that induce rejection by regulating Tregs. Next, we shall focus on investigating how to induce immune tolerance by regulating Tregs, DCs and other cells. Levine et al. demonstrated that Ex-527-mediated Sirt-1 inhibition enhanced the regulatory function of Foxp3^+^ Tregs by promoting *Foxp3* acetylation, thereby controlling T-cell-mediated rejection and improving kidney transplant survival and function in mice (163). Similar findings emerged in GVHD. Inhibiting Sirt-1 with Ex-527 or knocking out Sirt-1 suppressed T-cell allograft reactions and promoted Treg stability to control GVHD in mice (224). Sirt-1 deficiency reduced T-cell pathogenicity in individuals with graft-vs.-host disease by increasing *p53* acetylation in T cells and promoting iTreg stability. Moreover, treatment with Sirt-1 inhibitors reduced Tfh generation, B-cell activation, and plasma cell differentiation during cGVHD progression. Beyond inhibitor use, Iamsawat et al. observed that vitamin C supplementation in CD8^+^ iTreg cultures significantly increased DNA demethylation within conserved noncoding region 2 (CNCR2), maintaining elevated Foxp3 expression (13). Vitamin C-treated CD8^+^ iTregs suppressed pathogenic T-cell expansion and differentiation individuals with aGVHD while reduced thymic injury and B-cell activation in individuals with cGVHD. Similarly, Kasahara et al. also observed this phenomenon in murine graft-vs.-host disease and found that the conversion of iTregs into pathogenic exFoxp3 cells was reduced (225). Compared with untreated iTregs, vitamin C-treated iTregs suppressed GVHD symptoms more effectively. More importantly, vitamin C can also promote the conversion of human naive T cells into FOXP3^+^ iTregs; therefore, combining Treg therapy with vitamin C treatment holds promise for the future treatment of graft-vs.-host disease.

Regulatory-wise, small-molecule drugs such as Ex-527 face inherent challenges in conducting precise toxicological and dosing studies. For example, Sirtuin 1 is a multifunctional enzyme involved in various cellular processes; systemic inhibition may lead to unintended consequences beyond Treg regulation, affecting other immune cell populations and even non-immune tissues. Similarly, while vitamin C's role in promoting Treg differentiation is noteworthy, its non-nutritional pharmacological effects and the GMP compliance requirements for its therapeutic use necessitate rigorous investigation. Future challenges lie in developing more selective Sirt-1 variants or targeted delivery systems—such as coupling inhibitors to carriers that target immune cells, or utilizing biomaterials (such as hydrogel scaffolds) to achieve high local concentrations and low systemic concentrations of the drug—thereby maximizing therapeutic efficacy while minimizing systemic toxicity.

Modulation of non-coding RNAs can likewise induce peripheral immune tolerance. For miRNAs, Lu et al. showed that knocking down miRNA-146-5p increases TRAF6 expression in human tTregs *in vitro* (226). TRAF6 increases NF-*κ*B activation, which is crucial for tTreg function and Foxp3 protein and antiapoptotic gene expression, while downregulating proapoptotic gene expression. miR-146b knockdown increases the nuclear localization and expression of NF-*κ*B-regulated genes, which correlates with increased tTreg survival, proliferation, and suppressive function both *in vitro* and *in vivo*. Treatment with the miR-146-5p antagomir significantly increases the efficacy and persistence of human tTregs in a xenogeneic GVHD model. Zhang et al. demonstrated that miR-4281 efficiently and specifically upregulates Foxp3 expression by directly interacting with the TATA-box motif within the human *Foxp3* promoter (227). miR-4281 markedly accelerates the differentiation of human naive cells into induced Tregs (iTregs), which have immunosuppressive functions, and attenuates graft-vs.-host disease progression. For lncRNAs, Zhang et al. showed that lncRNA NEAT1 knockdown induces a tolerogenic phenotype in dendritic cells, thereby inducing immune tolerance (228). In a mouse model of cardiac transplantation, an infusion of NEAT1-knockdown DCs reduced inflammatory cell infiltration, suppressed T-cell proliferation, and increased the number of Tregs. For circRNAs, Wang et al. found that knocking down circFSCN1 expression induces tolerogenic DCs in a mouse heart transplant model, impairing their ability to activate T cells while increasing Treg generation (229). Treatment with these Tol-DCs reduces fibrosis and induces Treg production *in vivo*, preventing allogeneic immune rejection and prolonging allogeneic graft survival. Li et al. reported that knocking down circMAP2K2 expression decreased MHCII, CD40, and CD80 expression; weakened the ability of DCs to activate allogeneic naive T cells; and increased the number of Tregs (230). In a mouse cardiac transplantation model, treatment with circMAP2K2-knockdown DCs attenuated allogeneic immune rejection and prolonged allogeneic graft survival. Zhang et al. demonstrated that GDF15 induces the production of tolerance-inducing Tol-DCs by suppressing the activity of the circMalat1 and NF-*κ*B signaling pathways while upregulating IDO expression (231). The utilization of GDF15-DCs effectively prevented allogeneic immune rejection after cardiac transplantation in mice. Further studies have revealed that epigenetic mechanisms regulate transplant rejection through various mechanisms, thereby promoting transplant tolerance (232–242).

These studies collectively face regulatory challenges in translating from preclinical to clinical settings. For RNAi therapies, the delivery of nucleic acid drugs remains a major obstacle. Naked RNA molecules are prone to degradation and may trigger innate immune responses. Advanced delivery systems, such as lipid nanoparticles or engineered viral vectors, are needed to ensure efficient and targeted delivery to immune cells. While viral vector-based gene delivery offers improved targeting, it still faces challenges related to immunogenicity and safety assessment. Off-target binding of miRNAs or other ncRNAs to unintended mRNA targets may lead to unpredictable cellular responses. Given that non-coding RNAs often regulate gene expression by influencing chromatin structure and three-dimensional genomic architecture, designing ncRNAs that can precisely target specific chromatin regions and avoid off-target effects will be key to improving the precision of future therapies. For cell therapies, the core challenge lies in GMP-grade cell production, encompassing cell sourcing, expansion, quality control, and the standardization and validation of *in vitro* processing (e.g., antagomir or siRNA treatment) (243). Furthermore, although engineered Treg cells can enhance immunosuppressive function, their long-term survival in the body and ability to precisely target the graft still need to be improved; otherwise, this could lead to unintended immunosuppression and increase the risk of infection. Combining these technologies (RNAi + cell therapy) imposes heightened regulatory demands, requiring simultaneous demonstration of efficacy and safety for both components.

At the same time, striking a balance between immune suppression and host protection remains a future challenge. The core challenge lies in achieving sufficient immune tolerance to prevent rejection while not compromising the host's ability to resist infection or eliminate early-stage tumor cells. Excessive suppression of the immune system—particularly through the use of broad-spectrum small molecules or sustained ncRNA intervention—may lead to increased susceptibility to opportunistic infections and malignancies (243). Therefore, strategies to promote local immune tolerance—such as the targeted delivery of immunomodulatory factors—are crucial for mitigating systemic immunosuppression. Furthermore, with advances in spatial omics, single-cell multi-omics, and 3D genomics technologies, a precise analysis of chromatin conformation and epigenetic status within the local immune microenvironment of the graft will facilitate targeted interventions aimed at specific immune cells or chromatin regions, thereby inducing immune tolerance while maximizing the preservation of the body's normal immune surveillance functions (244).

Timely and appropriate intervention is also a strategy. However, the post-transplant immune response is highly dynamic and complex, with the immune microenvironment and epigenetic state varying at each stage. Identifying the optimal timing for epigenetic intervention to maximize the success rate of inducing tolerance and minimize the risk of rejection is a key challenge. In the future, single-cell ATAC-seq, Hi-C, Micro-C, and other multi-omics technologies can be combined to dynamically monitor the chromatin accessibility, three-dimensional genomic structure, and transcriptional regulatory networks of immune cells at different stages. This will enable the creation of epigenetic maps with higher spatiotemporal resolution, providing a theoretical basis for precisely selecting intervention windows and optimizing strategies for inducing immune tolerance (244).

## 7 Discussion

This review emphasizes the role of epigenetics in transplant rejection. We began by briefly surveying the historical development of epigenetics, its research trajectory in immunology and its extension to transplant rejection, alongside fundamental epigenetic mechanisms. Subsequently, we comprehensively described in detail the epigenetic mechanisms involved in ischemia‒reperfusion injury (IRI), transplant rejection and graft-vs.-host disease. Finally, given the heightened recent focus on epigenetics in transplant rejection therapy, we summarized relevant biomarkers and elucidated the epigenetic mechanisms to induce immune tolerance.

Rapid advances in epigenetics research have been propelled by bioinformatics applications, the establishment of extensive public datasets, and technological improvements in gene editing and sequencing. Current epigenetic research and applications for transplant rejection reveal that histone acetylation, miRNAs, and lncRNAs have been extensively investigated in the contexts of ischemia‒reperfusion injury and solid organ transplant rejection. Regarding graft-vs.-host disease (GVHD) occurring during hematopoietic stem cell transplantation, existing research has primarily focused on acute GVHD (aGVHD) and has already yielded a relatively comprehensive body of findings involving a wide range of epigenetic regulatory mechanisms; but conducting more in-depth mechanistic and clinical studies would be highly beneficial.

Despite recent advancements, significant knowledge gaps and technical limitations persist in our understanding of epigenetic regulation in transplant immunity. Current research exhibits a notable lack of comprehensiveness and balance, with a disproportionate focus on a limited set of epigenetic mechanisms. Crucially, the roles of other key epigenetic regulators, including DNA methylation, chromatin remodeling, and histone methylation, remain underexplored in the context of transplant immunity. This deficit is particularly pronounced in specific rejection scenarios, such as chronic GVHD (cGVHD), where dedicated investigation is urgently needed. Moreover, the intricate interplay among epigenetic mechanisms—where DNA methylation can influence histone modifications, and non-coding RNAs orchestrate both—has not been adequately elucidated. The synergistic contributions of these interconnected pathways to transplant immunity are largely unknown, necessitating further research to construct a comprehensive network model that captures these complex interactions.

Furthermore, emerging fields and cutting-edge technologies remain significantly underrepresented. First, most current studies rely on bulk analyses of PBMCs or whole-graft biopsies, which are inherently confounded by cell-type heterogeneity; thus, observed epigenetic alterations may merely reflect shifting immune cell proportions rather than cell-intrinsic regulatory programs. To address this, integrating advanced single-cell (e.g., scATAC-seq, scMe-seq) and spatial epigenomics is urgently needed to map cell-type-specific epigenetic landscapes within the graft microenvironment. Second, existing literature is largely limited by “snapshot” observations, lacking longitudinal clinical correlation to predict long-term graft survival or chronic rejection. Future efforts must prioritize prospective, multi-center cohorts with longitudinal follow-up and standardized biosampling, directly correlating dynamic epigenetic profiles with real-world clinical endpoints to bridge the gap between bench and bedside.

Although much of the current evidence is derived from other transplant settings, emerging studies have demonstrated an important role for epigenetic regulation in heart transplantation. Epigenetic mechanisms contribute to ischemia–reperfusion injury, acute rejection, chronic rejection, and cardiac allograft vasculopathy by regulating immune responses, cardiomyocyte survival, and vascular remodeling. Future studies integrating human endomyocardial biopsy samples with single-cell and spatial epigenomic technologies will further clarify cardiac-specific epigenetic mechanisms and accelerate the translation of epigenetic biomarkers and therapeutic targets into clinical practice.

Currently, the primary clinical approach to suppressing rejection relies on immunosuppressive therapy, which typically involves the use of drugs such as cyclosporine, tacrolimus, and mycophenolate mofetil (245–247). Moreover, a series of drugs targeting DNA methylation and histone acetylation have been developed and are already widely used clinically in oncology (248–251). Despite extensive research on epigenetic modifications in transplant rejection, their direct clinical translation for solid organ transplantation is limited, evidenced by a paucity of clinical trials, with the majority of investigations confined to rodent models (252–255). Conversely, for hematopoietic stem cell transplantation, numerous clinical trials have substantiated the therapeutic efficacy of epigenetic drugs, though their widespread clinical implementation necessitates further development (256–259). Furthermore, epigenetic drugs are likely to be used in combination with existing immunosuppressive regimens in the future. Therefore, further research is needed on drug-drug interactions, cumulative toxicity, and how to optimize these combinations to achieve tolerability while reducing the dosage of traditional immunosuppressants. Therefore, further investigation into the role of epigenetic drugs in human transplant rejection is critically important.

In summary, epigenetics represents a promising field for the diagnosis and treatment of posttransplant rejection. Moving forward, we aim to further explore the molecular mechanisms by which epigenetics regulates rejection responses and identify additional strategies for the prevention and treatment of transplant rejection to better guide clinical care, ultimately leading to longer-term survival and improved quality of life for organ transplant recipients.
